# Rebalancing TGF-β/PGE_2_ breaks RT-induced immunosuppressive barriers by enhancing tumor-infiltrated dendritic cell homing

**DOI:** 10.7150/ijbs.87867

**Published:** 2024-01-01

**Authors:** Ziqi Zhou, Qianqian Zhou, Jing Zhao, Xiaorong Hou, Junfang Yan, Xiansong Sun, Zhiwei Yang, Jiabin Ma, Fuquan Zhang, Linsheng Zhan, Ke Hu

**Affiliations:** 1 Department of radiation oncology, Peking Union Medical College Hospital. Chinese Academy of Medical Sciences and Peking Union Medical College, Beijing, People's Republic of China.; 2 Institute of Health Service and Transfusion Medicine, Tai Ping Road, Beijing 100850, People's Republic of China.; 3 Department of Oncology, Beijing Shijitan Hospital, Capital Medical University, Beijing, People's Republic of China.; 4 State Key Laboratory of Complex Severe and Rare Diseases, Peking Union Medical College Hospital, Chinese Academy of Medical Science and Peking Union Medical College, Beijing, People's Republic of China.

**Keywords:** dendritic cell, radiotherapy, cervical cancer, TGF-β

## Abstract

A better understanding of how tumor microenvironments shape immune responses after radiotherapy (RT) is required to improve patient outcomes. This study focuses on the observation that dendritic cells (DCs) infiltrating irradiated cervical tumors are retained in transforming growth factor (TGF)-β-abundant regions. We report that TGF-β secretion from cervical cancer cells was increased by irradiation in a dose-dependent manner and that this significantly suppressed the expression of allostimulatory markers and Th1 cytokines in DCs. To investigate further, we blocked the TGF-β signal in DCs and observed that RT had a dose-dependent immune-promoting effect, improving DC maturation. This suggested that proinflammatory mediators may also be induced by RT, but their effects were being counteracted by the simultaneously increased levels of TGF-β. Prostaglandin E2 (PGE_2_), a proinflammatory molecule, was shown to be one such mediator. Adjusting the TGF-β/PGE_2_ ratio by inhibiting TGF-β rebooted RT-induced DC cytoskeletal organization by stimulating myosin light chain (MLC) phosphorylation. Consequently, the homing of intra-tumorally infiltrated DCs to tumor-draining lymph nodes was enhanced, leading to the induction of more robust cytotoxic T cells. Ultimately, rebalancing the TGF-β/PGE_2_ ratio amplified the therapeutic effects of RT, resulting in increased intra-tumoral infiltration and activation of CD8^+^ T cells, and improved tumor control and overall survival rate in mice. DC depletion experiments verified that the improvement in tumor control is directly correlated with the involvement of DCs via the PGE_2_-MLC pathway. This study emphasizes the importance of maintaining a balanced cytokine environment during RT, particularly hypofractionated RT; and it is advisable to block TGF-β while preserving PGE_2_ in the tumor microenvironment in order to better stimulate DC homing and DC -T priming.

## Introduction

Cervical cancer is the fourth most frequently diagnosed malignant tumor and the fourth leading cause of cancer deaths in females, with an estimated 604,000 new cases and 342,000 deaths worldwide in 2020 1. Currently, curative-intent radiotherapy (RT) plus brachytherapy is the mainstay of first-line treatment for locally advanced cervical cancer. A clear dose-dependent relationship between RT and survival rate of patients with cervical cancer is described by the EMBRACE (European Study on MRI-guided Brachytherapy in Locally Advanced Cervical Cancer) studies 2. Additionally, as image-guided RT techniques have become the gold standard for the treatment of cervical cancer, the pattern of relapse is primarily systemic. Of all patients with treatment failure, 21% had pelvic failure, 57% had distant failure, and 23% had both pelvic and distant failure 3. Unlocking the mechanisms behind the systemic failure of cervical cancer and identifying new therapeutic targets are critical for improvement of treatment outcomes. Effective treatments to eradicate micrometastases in the para-aortic lymph nodes (LNs) and distant organs are needed in addition to the current standard treatment protocol to increase disease control and improve survival.

Increasing evidence has demonstrated that RT can kill cancer cells while simultaneously remodeling the immune context of the tumor microenvironment (TME). From the perspective of immuno-oncology, RT is a double-edged sword that gives rise to both immunogenic and immunosuppressive effects in tumors 4. Hypo-fractionated RT (hypo-RT) can modulate tumor neoantigen expression, trigger the release of proinflammatory mediators, and drive immune cell infiltration 5. Radiation-mediated immune suppression has been linked to reduction of anti-inflammatory mediators, tumor infiltration of immunosuppressive cell types, and the exhaustion of effector immune cells 6. Altogether, RT potentially elicits multiple layers of immune responses depending on the tumor biology, induction of immunosuppressive signals, and immune cell infiltration post-RT. Consequently, the translation of preclinical investigation results to their clinical application is more convoluted than anticipated which underlines the need to decode molecular and cellular mechanisms induced by RT 5.

In recent years, the emergence of immune checkpoint inhibitors, including those targeting PD-1, PDL-1, and CTLA4 has promoted the combination of RT with immunomodulatory biological agents that increase the therapeutic benefits post-RT 7. The refinement of this strategy requires not only the manipulation of the balance of pro- and anti-tumor mediators but also thoughtfully designed studies encompassing variations in the dose and fractionation schedules of RT. As one of the most studied and powerful immune-regulatory mediators, transforming growth factor (TGF)-β exerts profound effects on major cellular components of both the innate and adaptive immune systems 8. There is now overwhelming evidence to support the role of a TGF-β-rich TME in suppressing anti-tumor Th1 responses. TGF-β can act directly on dendritic cells (DCs), the most potent antigen-presenting cells, by downregulating allostimulatory markers on their surface, converting immature DCs to the immunosuppressive phenotype 9, and contributing to suppressive effects of T cell activation 10. However, compared to the use of immune checkpoint inhibitors targeting PD-1, PDL-1, or CTLA4, therapies based on inhibiting the TGF-β pathway have progressed more slowly in oncology. This is largely due to the complex biology of the TGF-β family and the context-dependent nature of their function 11. All variables including the tumor histological type and progression stage, monotherapy versus combined strategies, and the dosage and/or delivery regimens, significantly affect therapeutic outcomes. Benefiting from in-depth basic studies on TGF-β in relation to pancreatic, breast, and prostate cancers, drugs targeting TGF-β signaling combined with RT or chemotherapy have reached phase I-II clinical trials 12-15. In patients with unresectable pancreatic cancer, galunisertib, a TGF-β receptor inhibitor, combined with chemotherapy showed improved overall survival compared with chemotherapy alone 16. However, distinct from the above-mentioned tumors, TGF-β targeting therapy is seldomly explored in cervical cancer clinically. The lack of clinical trial data is particularly relevant to the fact that many aspects of TGF-β impacting radiation-induced immune system changes in cervical cancer are insufficiently explored. Key issues include whether cervical cancer could benefit from TGF-β inhibition, whether a synergistic effect can be generated from combining TGF-β inhibition with RT, the choice of RT regimen (especially the RT dose) that maximizes efficacy, and the mechanisms involved is still to be addressed.

Our preliminary study found that in tissue specimens from patients with cervical cancer who received radical RT plus brachytherapy, the DCs were highly retained within TGF-β-abundant regions. This phenomenon prompted us to investigate whether it was RT-induced-TGF-β upregulation that caused this retention of DCs within cervical tumors and to determine the significance of modulating TGF-β while conducting RT. Herein, we demonstrated for the first time that RT induced a TGF-β/PGE_2_ imbalance, especially hypo-RT, was a limiting step for tumor-infiltrated DC homing. We report that inhibition of TGF-β is sufficient to overcome the RT-related immunosuppressive effects and improve prognosis. The findings of our study advance the understanding of radio-immunology and support the use of RT in combination with TGF-β inhibitors for the treatment of cervical tumors.

## Results

### Tumor-infiltrating DCs concentrate in TGF-β-abundant regions

According to the National Comprehensive Cancer Network guidelines, the treatment strategy for patients with locally advanced cervical cancer (LACC) is definitive concurrent chemoradiotherapy (CCRT) consisting of external beam RT with concomitant platinum-based chemotherapy and brachytherapy. Considering that the 5-year survival rate of patients remains 70%, adjuvant hysterectomy has been used after CCRT to improve survival in certain patients 17, 18. But the benefits of adjuvant hysterectomy after definitive CCRT is controversial and requires further investigation. In the past 15 years, the gynecological group of Peking Union Medical College Hospital (PUMCH) selectively chose patients to undergo extra fascial hysterectomy after CCRT and retrospectively compared the prognosis of patients with those receiving the current standard care 19. The paraffin slides we used as post-RT samples in this study were obtained from paraffin wax blocks of eight LACC patients with FIGO stage IB2, IIA2, and IIB who were treated with CCRT and underwent an extra fascial hysterectomy at PUMCH between January 2005 and 2020. The RT regimen Supplementary Table 1 was via an external beam delivering 41.4-50.4 Gy in 23-28 fractions followed by brachytherapy (30-36 Gy in 5-6 fractions). Tumor sections from four patients with cervical cancer who receiving no irradiation were used as controls. The informed consent was waived due to its retrospective nature. All study protocols involving humans were approved by the Institutional Review Board (Approval No: SK1210).

**Figure 1:**
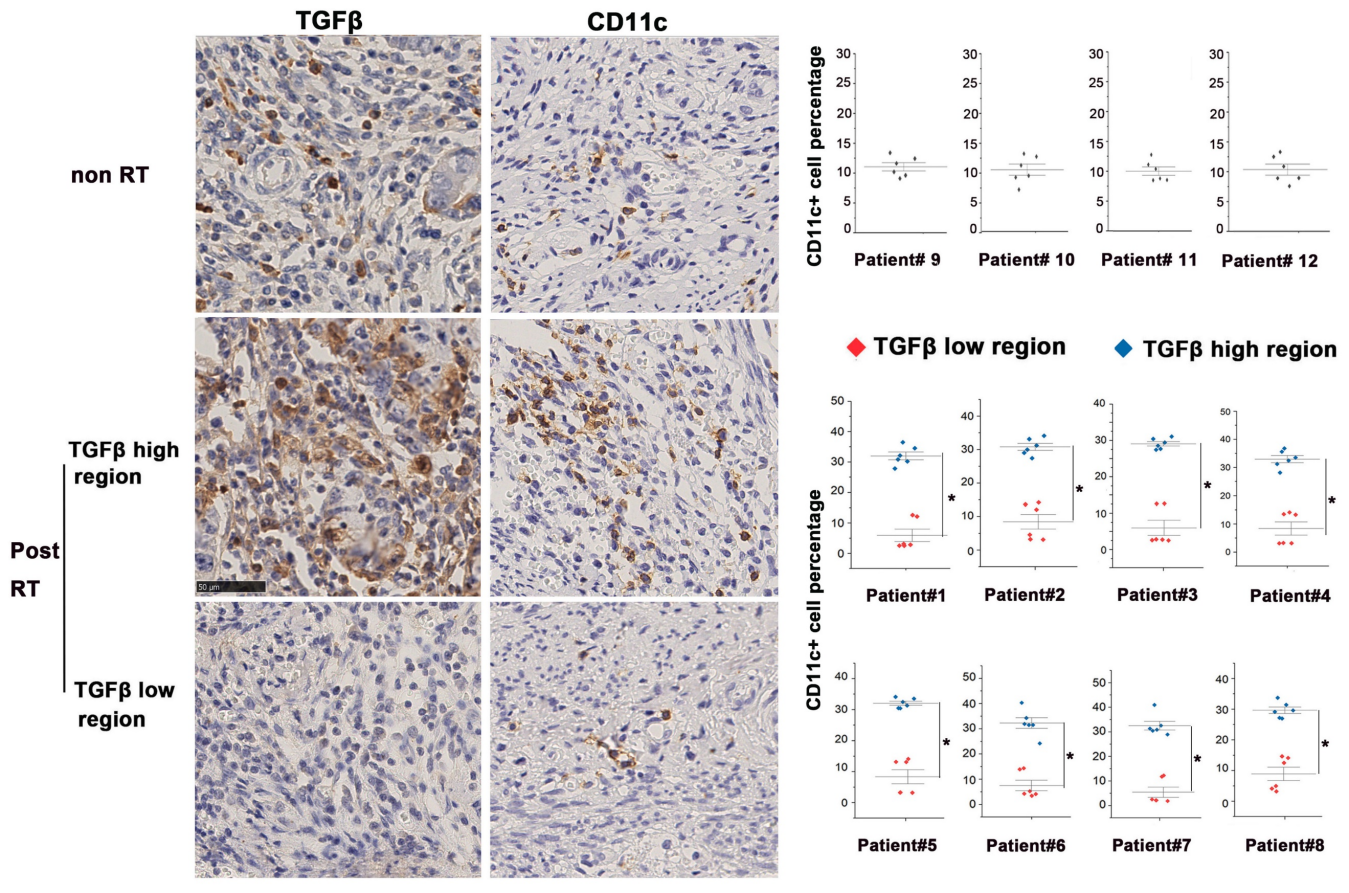
Altered homeostasis of tumor-infiltrating DCs in the presence of high levels of TGF-β. Immunohistochemical staining of TGF-β and CD11c in tissue samples of eight cervical cancer patients after radical RT (chemoradiotherapy plus brachytherapy). The first row displayed representative images of non-RT tumors. The second and third rows displayed the profile of TGF-β-enriched areas and TGF-β-sparse areas. Quantification of staining in clinical samples was performed using Image J; *p < 0.05.

In this study, we compared post-RT samples with non-irradiated cervical cancer samples to investigate the TME post-RT. Tissue slices obtained from continuous sectioning of blocks were stained with anti-TGF-β and anti-CD11c^+^ (a specific marker of human DCs) antibodies for analysis. In the non-irradiated cervical cancer biopsies, TGF-β was moderately expressed and evenly distributed. By contrast, TGF-β expression of irradiated tissues was highly heterogeneous, with the co-existence of both high- and low-expression regions. We found that CD11c^+^ DCs were also unevenly distributed in irradiated tissues and more densely distributed in TGF-β-abundant regions, whereas the distribution of DCs in low TGF-β expression regions was scant (Fig. 1). Furthermore, bioinformatic analysis based on samples in the TIMER database also demonstrated that DCs accumulated in TGF-β high-expression tumors, which was consistent with our findings Supplementary Fig. 1. These results demonstrate that RT causes significant changes in TGF-β expression and DC distribution in the TME of cervical cancer.

### RT induced a contradictory effect on DC maturation determined by TGF-β signaling

**Figure 2:**
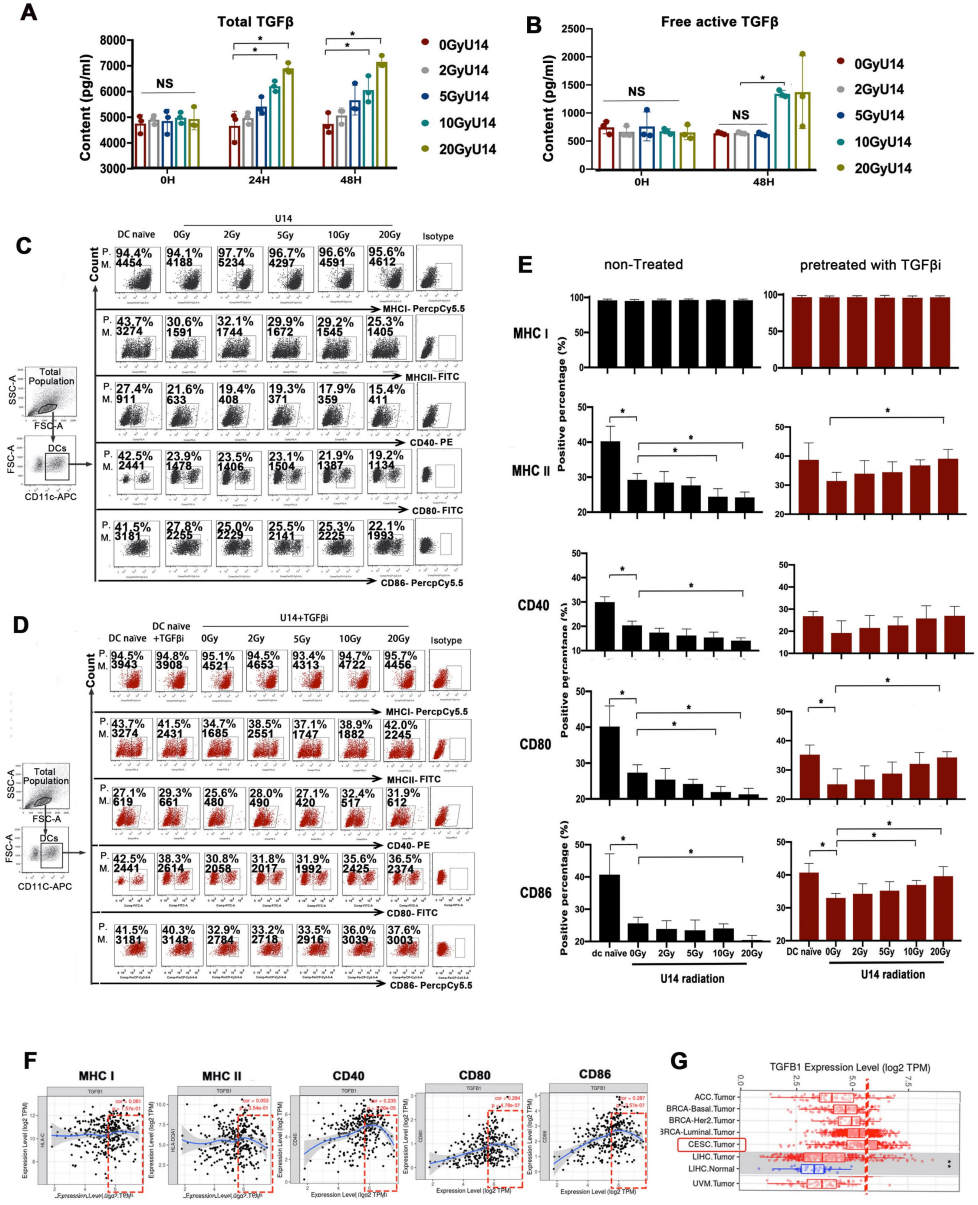
RT induced a contradictory effect on DC function determined by TGF-β signaling. (A) The secretion levels of total TGF-β in the supernatant of irradiated U14 cells; N= 3, *p < 0.05. (B) The secretion levels of free active TGF-β in the supernatant of irradiated U14 cells; N= 3-5, *p < 0.05. (C) The supernatant of irradiated U14 cells was collected 24 h after radiation exposure. DCs were co-incubated with the supernatant for 24 h and the DC phenotype was analyzed by flow cytometry. (D) The supernatant of irradiated U14 cells was cocultured with DCs pretreated with TGF-β receptor antagonist (TGF-βi: galunisertib 50 μM), and the DC phenotype was analyzed. The expression of CD40, CD80, CD86, MHCI, and MHCII in CD11c^+^ cells was detected with flow cytometry. P., positive percentage; M., mean fluorescence intensity. (E) Bar graphs and statistical analysis of the FACS results; N= 3-5, *p < 0.05. All above data are representative results from two or three independent experiments. (F) Correlation analysis of *TGFB1* expression and the expression of marker genes of infiltrating DCs in cervical cancer according to the TIMER database. (G) The level of *TGFB1* expression in different tumor types according to the TCGA database in TIMER (CESC: cervical squamous cell carcinoma).

To investigate the effects of RT on TGF-β expression and its downstream effects on DCs, we exposed U14 cervical cancer cells (derived from mice) and SiHa cervical cancer cells (derived from humans) to X-rays at doses of 2, 5, 10, and 20 Gy. After 24 h of culture, cellular secretions were collected for analysis. The levels of both total TGF-β (Fig. 2A and Supplementary Fig. 2) and the free active form of TGF-β (Fig. 2B), measured in the supernatants of irradiated cell lines, were increased in a dose-dependent manner. Subsequently, we added the supernatant to DCs and evaluated the effect on their phenotype. Compared to naïve DCs, DCs challenged with tumor supernatant exhibited significantly suppressed expression of MHC II, CD40, CD80, and CD86 (Fig. 2C). Importantly, the suppressive effects on DCs increased with increasing doses of RT. Blocking of TGF-β receptor signaling with galunisertib greatly alleviated the suppression of DCs, implicating TGF-β in the supernatant as a key mediating factor (Fig. 2D).

Furthermore, bioinformatic analysis based on data from the TIMER database also revealed a correlation between TGF-β expression in cervical cancer tissues and the maturation of tumor-infiltrating DCs (Fig. 2F). The analysis showed a U-shaped curve, indicating that beyond a certain concentration (threshold) of TGF-β, the dose-response relationship shifted from positive to negative. Further analysis based on RNA-seq data from The Cancer Genome Atlas (TCGA) indicated that the average baseline of TGF-β mRNA expression in cervical and endocervical cancer (CESC) was relatively close to the threshold (Fig. 2G). The results from Fig. 2F-G together suggest that with the RT-induced increase in TGF-β secretion (beyond the baseline level) may downregulate the expression of allostimulatory markers on DCs in a dose-dependent manner. This speculation is consistent with the results presented in Fig. 2C-E. However, this preliminary inference requires further investigation.

From Fig. 2E, an interesting phenomenon emerges: the effects of RT on DC phenotype are completely opposite depending on whether TGF-β signaling is blocked or not. Without TGF-β signaling blockage, RT dose-dependently enhances tumor-induced DC suppression (Fig. 2C and E). However, when TGF-β signaling is inhibited, the suppressed expression of MHC II, CD40, CD80, and CD86 on DCs by tumor supernatant is substantially attenuated. Notably, these effects are then recovered in an RT-dose-dependent manner, with the highest levels observed in the DC_20Gy/TGFβi/U14_ group (Fig. 2D and E). Similar trends can be observed in the SiHa tumor cell line, where the most matured phenotype is observed in the DC_20Gy/TGFβi/SiHa_ group Supplementary Fig. 3. These results suggest that RT has both immunostimulatory and immunosuppressive effects, with the stimulatory effects being antagonized by the upregulation of suppressive mediators, such as TGF-β. Therefore, the stimulatory effect was only apparent when the suppressive pathway is blocked.

### RT-induced PGE_2_ elevation reboots tumor-suppressed DC maturation

As mentioned above, key proinflammatory mediators should also be induced by RT to reboot DC maturation though their effects were antagonized by the simultaneously upregulated TGF-β. Among the known mediators that effectively modulate the immunosuppressive TME in a beneficial manner, high mobility group box 1 protein (HMGB1) and ATP, are widely recognized as representatives of damage-associated molecular patterns. These molecules play a crucial role in enhancing antigen uptake by DCs, promoting their maturation, and facilitating antigen presentation, ultimately leading to the activation of cytotoxic T cells. PGE_2_, on the other hand, is a versatile cytokine that can either positively or negatively influence various aspects of DC function, depending on the specific microenvironment. Hence, we assessed HMGB1, ATP, and PGE_2_ as potential key proinflammatory mediators, because they have been implicated in radiation-induced immune system activation 20, 21.

**Figure 3:**
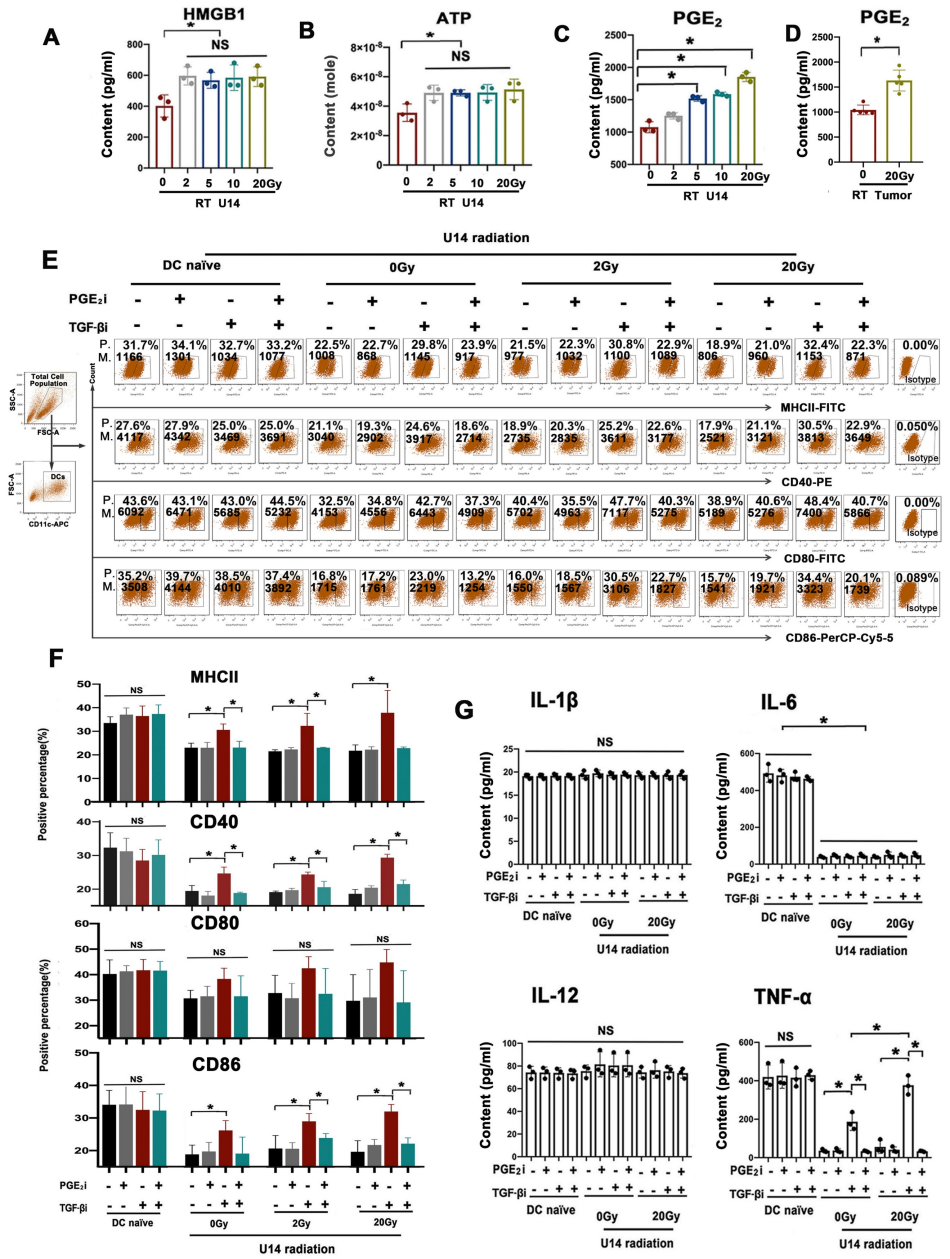
Rebalancing TGF-β/PGE_2_ towards PGE_2_ polarization promoted the immunostimulatory effect of irradiated U14 cells on DC phenotype and TNF-α secretion. The secretion levels of HMGB1 (A), ATP (B), and PGE_2_ (C) in the supernatant of radiated U14; N = 3, *p < 0.05. (D) The level of PGE_2_ in the TME of tumor-bearing mice was evaluated by ELISA; N = 5, *p < 0.05. (E) DCs pretreated with PGE_2_i or TGF-βi were co-incubated with the supernatant for 24 h, then the DC phenotype was measured by flow cytometry. P., positive percentage; M., mean fluorescence intensity. (F) Bar graphs and statistical analysis of the FACS results. N = 3, *p < 0.05. (G) The secretion of the proinflammatory cytokines of IL-1β, IL-6, IL-12p70, and TNF-α was measured by ELISA; N = 3, *p < 0.05. All above data are representative results from two or three independent experiments.

As depicted in Fig. 3A-C, the levels of HMGB1, ATP, and PGE_2_ in U14 cell supernatants significantly increased after radiation. However, only PGE_2_ levels increased in a dose-dependent manner (Fig. 3C and Supplementary Fig. 2). The U14 tumor tissue from tumor-bearing mice exhibited a significant increase in PGE_2_ levels after hypo-RT (Fig. 3D). This was consistent with the trend of RT-enhanced DC maturation (Fig. 2D). To investigate whether RT-induced PGE_2_ elevation was critical for DC maturation, a PGE_2_ receptor antagonist (AH6809) was added to block the PGE_2_ signal of DCs before the cells were challenged by irradiated U14 supernatant, with or without TGF-β signal blocking. Neither PGE_2_i nor TGF-βi directly caused significant changes in the DC phenotype Supplementary Fig. 4 and 5). For irradiate of U14 cells, RT doses of 2 and 20 Gy were chosen as representative of conventional fractionated RT and hypo-RT, respectively. As shown in Fig. 3E and F, and Supplementary Fig. 3, tumor secretions significantly impaired the expression of CD40, CD80, CD86, and MHCII. Irradiation, especially hypo-RT, combined with TGF-β signaling inhibition largely relieved this suppression. However, simultaneous PGE_2_ inhibition dramatically attenuated the upregulated expression of costimulatory molecules brought about by RT and TGF-β blocking. Furthermore, a similar tendency was observed for the secretion of Th1 cytokines by DCs (Fig. 3G). IL-6 and TNF-α levels were substantially suppressed by U14 secretions. RT combined with TGF-β signaling blockade significantly augmented the secretion of TNF-α while blocking PGE_2_ overrode this effect. All of those data confirmed PGE_2_ was indeed a critical proinflammatory mediator induced by RT to reboot cervical cancer cell-suppressed DC maturation.

### Rebalancing TGF-β/PGE_2_ signaling promoted cytoskeletal reorganization and chemotaxis of DCs

**Figure 4:**
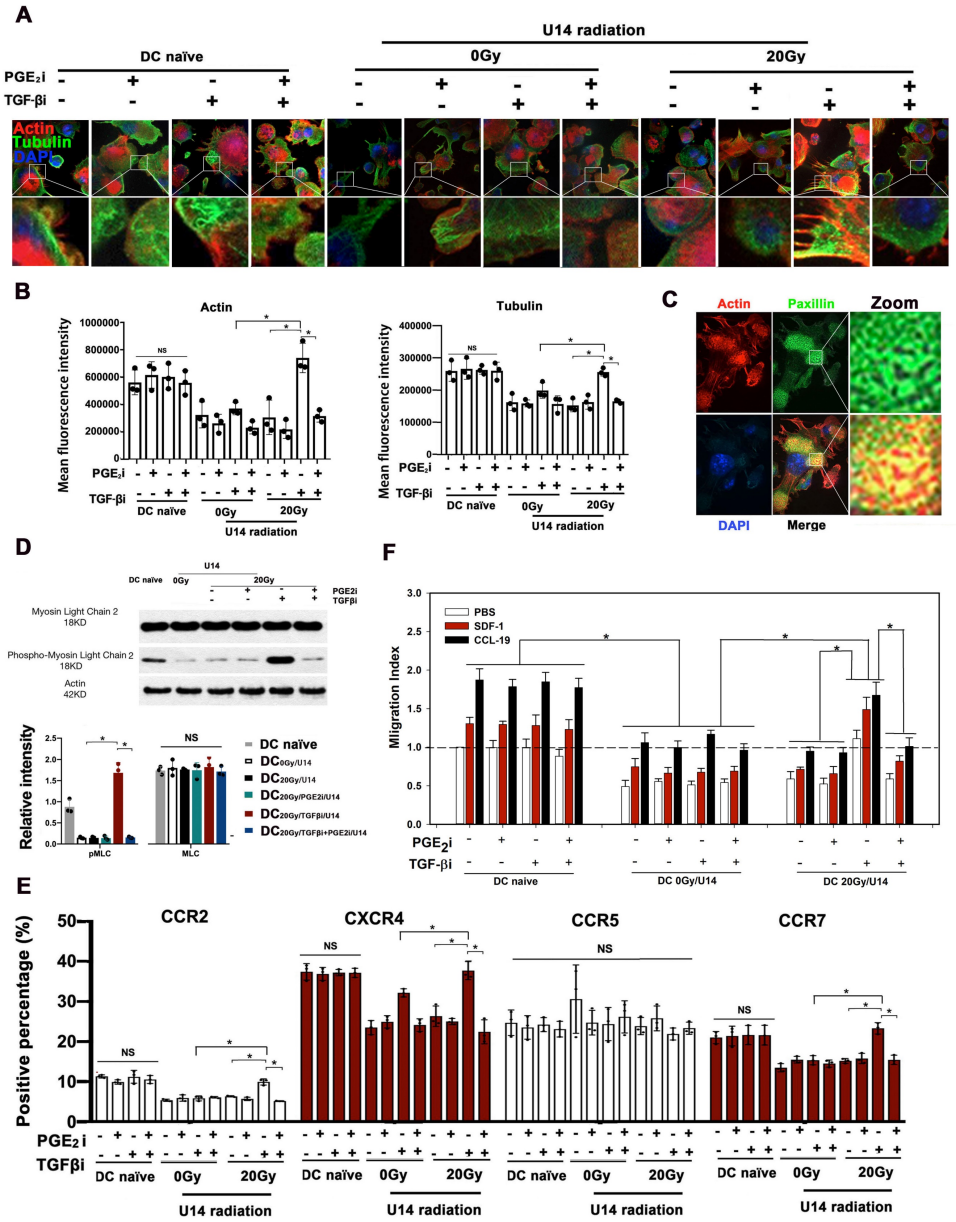
Rebalancing TGF-β/PGE_2_ towards PGE_2_ polarization promoted DC cytoskeletal reorganization. (A) U14 cells were incubated with tumor secretions for 24 h post-RT. The supernatant was collected and cocultured with DCs in the presence or absence of TGF-β/PGE_2_ receptor antagonists. The organization of microfilaments and microtubules in DCs was observed by staining the F-actin and β-tubulin. (B) Statistical analysis of the mean fluorescence intensity of microfilaments and microtubules; N = 3 views; *p < 0.05. (C) The formation of focal adhesions on DC_20Gy/TGF-βi/ U14_s stained with an anti-paxillin antibody. (D) Activation of the MLC signaling pathway in DCs was assessed by western blotting. The results are presented as the relative intensity of the protein bands from N = 3 replicates; *p < 0.05. (E) The chemokine receptor expression of DCs after different treatments was detected with FACS; N = 3; *p < 0.05.(F) Migration index was measured in transwell experiments using SDF-1 and CCL-19 as respective ligands for CXCR4 and CCR7; N = 5, *p < 0.05. All above data are representative results from two or three independent experiments.

Microfilaments and microtubes form the cytoskeletal intracellular network responsible for cell migration (22. To investigate whether RT-induced-TGF-β/PGE_2_ changes affect DC migration, DCs were pulsed by secretions from non-irradiated or 20 Gy irradiated U14 cells and stained with phalloidin (F-actin visualization) and β-tubulin antibody (microtubules visualization). Secretions from non-irradiated or irradiated U14 cells, dramatically inhibited DC cytoskeletal organization, as demonstrating by very weak staining of both F-actin and β-tubulin (Fig. 4A-B). Blocking TGF-β signaling rescued the inhibited cytoskeletal network in DC_20Gy/TGFβi/U14_ but not in DC_0Gy/TGFβi/U14_. Notably, combined blockade of PGE_2_ and TGF-β signaling greatly reduced the stimulatory effect of the TGF-β signal blockade. These results indicated that hypo-RT played a similar promoting role in DC cytoskeletal organization as in DC maturation mainly through the PGE_2_ signaling pathway, an effect that was suppressed by the presence of TGF-β. In the microfilament-concentrating region of DC_20Gy/TGFβi/U14_, staining using an anti-paxillin antibody (Fig. 4C) showed typical focal adhesion, confirming the functional competence of the cytoskeletal network induced by rebalancing TGF-β/PGE_2_ signaling. Myosin light chain (MLC), the essential component of non-muscle myosin II A, plays an indispensable role in focal adhesion assembly and cytoskeletal organization 23. MLC phosphorylation, mediated by MLC kinase determined its activity. As shown in Fig. 4D, the level of phosphorylated MLC in DC_20Gy/TGFβi/U14_ cells was higher than that in the DC_0Gy/U14_ or DC_20Gy/TGFβi+PGE2i/U14_ treated groups. Thus, hypo-RT combined with TGF-β blockade releases the cervical cancer-induced suppression of DC cytoskeletal organization via the PGE_2_-MLC pathway.

DC migration is a tightly regulated process, depending on both cytoskeletal function and chemokine guidance. We found that secretions from irradiated or non-irradiated U14 cells significantly suppressed the expression of CCR2, CXCR4, and CCR7 on DCs, but CCR5 was not affected by the treatment. The suppression of these chemokine receptors could be prevented in the DC_20Gy/TGFβi/U14_ group by blocking TGF-β signaling, but this manipulation was less effective in DC_0Gy/TGFβi/U14_ group, resulting in only partial recovery of CXCR4 expression (Fig. 4E). Furthermore, results from transwell experiments using SDF-1 and CCL-19 as respective ligand for CXCR4 and CCR7 confirmed the changes in chemokine receptor levels on DCs (Fig. 4F). Collectively, these data demonstrate that hypo-RT combined with rebalancing of TGF-β/PGE_2_ signaling promotes cytoskeletal reorganization and chemotaxis of DCs.

### The balance of TGF-β and PGE_2_ determined the *in vivo* homing ability of DCs

**Figure 5:**
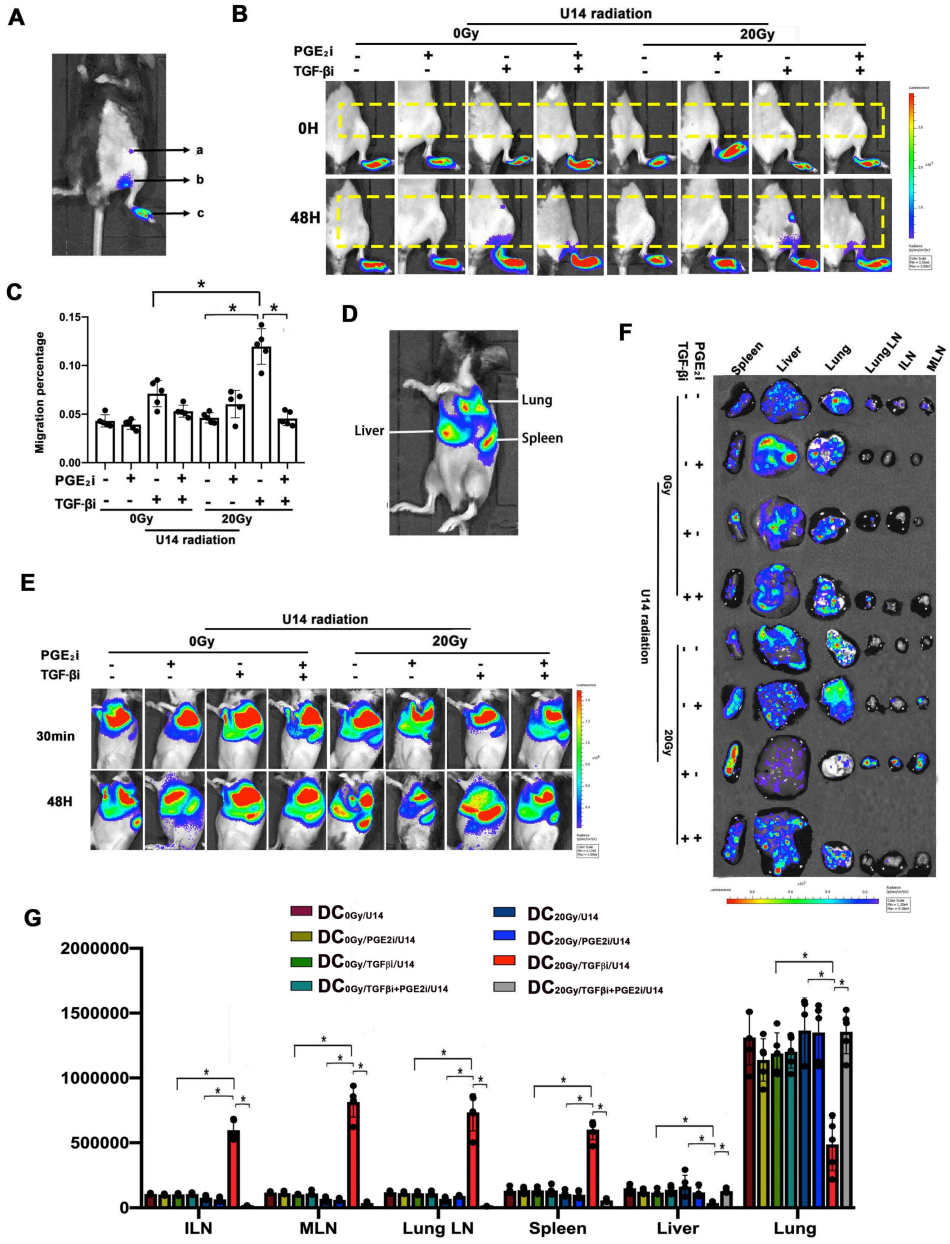
The balance of TGF-β and PGE_2_ determined the* in vivo* homing ability of DCs. (A) Annotation of the light signal from Fluc^+^ DCs after footpad injection. The signal intensity represents the number of DCs that migrated to the region "a" inguinal LNs (ILNs), region "b" popliteal LNs, and region "c" residual DCs in the footpad. To quantify migrating capability, the following formula was utilized: migration percentage = (a + b) / (a + b + c) × 100%. (B) Fluc^+^ DCs (pretreated with PGE_2_i/TGF-βi) were cocultured with the supernatant of irradiated U14 for 24 h and were then injected subcutaneously into the footpad of wild-type C57BL/6 mice. The dynamic migration process was imaged. (C) Statistical analysis of regional DC migration; N = 5 in each group; *p < 0.05. (D) Annotation of the light signal of Fluc^+^ DCs after intravenous injection. (E) Fluc^+^ DCs (pretreated as described) were injected intravenously into the tail vein, and the dynamic migration process of DCs was detected. (F) Tissue distribution of Fluc^+^ DCs. (G) DC migration to tissues. The total amount of Fluc activity was normalized to the weight of the individual tissue; N = 5 in each group; *p < 0.05. All above data are representative results from three independent experiments.

Next, the *in vivo* homing of DCs was examined non-invasively by bioluminescence imaging. Given that local LNs are the most common homing sites of intra-tumoral DCs and the location of T cell activation, we utilized a footpad injection model to evaluate the homing ability of local residual Fluc^+^ DCs to reach LNs *in vivo*. U14 cells were treated with a hypo-RT dose of 20 Gy in these experiments, which could be approximately matched to the high dose rate brachytherapy that cervical cancer patients received 24. DCs treated with or without TGF-β/PGE_2_ inhibitors were pulsed with U14 secretions and then injected into the footpad of wild-type C57BL/6J mice followed by dynamic imaging analysis. Blocking the TGF-β signal (DC_20Gy/TGFβi/U14_) significantly promoted the homing of DCs pulsed by tumor secretions, whereas simultaneous blockade of PGE_2_ signaling (DC_20Gy/TGFβi+PGE2i/U14_) overrode this stimulatory effect, resulting in most DCs remaining localized in the footpad (Fig. 5 B and C). Thus, hypo-RT combined with rebalancing of TGF-β/PGE_2_ signaling promoted DC homing to local LNs.

Recirculation of resident DCs has been demonstrated to initiate systemic immune responses 25, 26. Fluc^+^ DCs (3 × 10^6^) of various treatments were intravenously injected into wild-type C57BL/6J mice to mimic blood-circulating DCs. Fig. 5D shows the signal from Fluc^+^ DCs after intravenous infusion. As shown in Fig. 5E, a similar distribution pattern was observed immediately after the injection: most DCs accumulated in the lung, mainly due to mechanical trapping by pulmonary capillaries rather than active adhesion. The differences in trafficking appeared 48 h post-infusion, and the majority of DCs in mice that received only RT or TGFβi were still detectable in the lung at 48 h. Cells in the DC_20Gy/TGFβi/U14_ group migrated the fastest, and most of these cells had already traveled from the lung and were concentrated in the spleen, inguinal LN (ILN), mesenteric LN, and lung LN at 48 h. Concurrent inhibition of PGE_2_ overrode this effect (Fig.5E-G). These results corroborate the findings of DC cytoskeletal rearrangement and chemotaxis (see Fig. 4) and demonstrate that hypo-RT combined with rebalancing of TGF-β/PGE_2_ signaling towards PGE_2_ polarization enhances both local and circulating DC homing to lymphoid tissues.

### Rebalancing TGF-β/PGE_2_ contributed to DC-T cell activation

**Figure 6:**
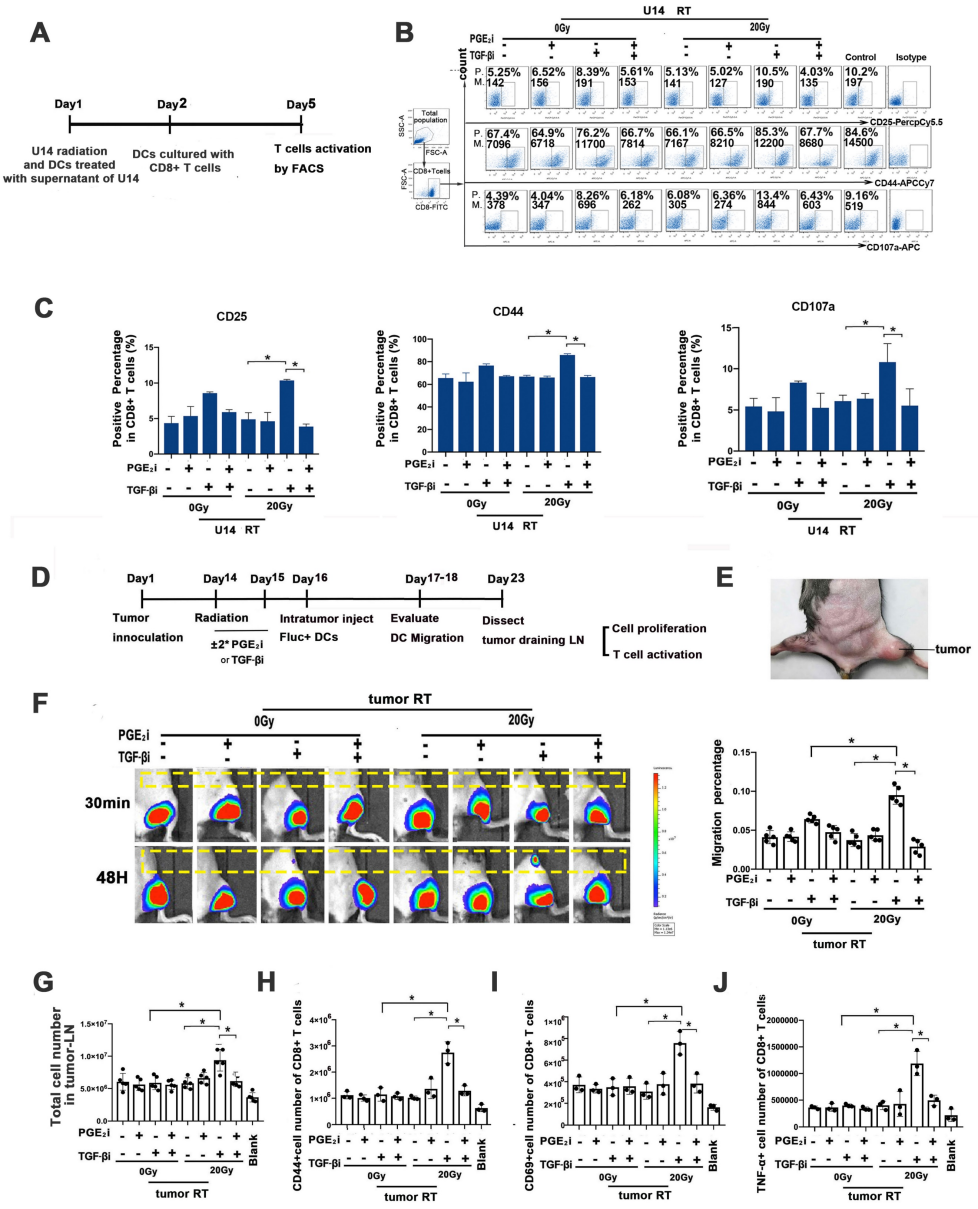
Rebalancing TGF-β/PGE_2_ contributed to T cell activation. (A) Flowchart of the *in vitro* experiment. DCs of virous treatments incubated with OVA-I (1 µg/ml); CD8^+^ T cells were isolated from the spleen of OT-I mice using CD8^+^ T cell sorting magnetic beads. Then DC and T cells were co-cultured at a 1:5 ratio for three days. After that T cells were stained for FACS. (B) *In vitro* T-cell activation was measured by flow cytometry (P., positive percentage; M., mean fluorescence intensity). (C) Bar graphs of the results of FACS experiments; N = 3, *p < 0.05. (D) Flowchart of the *in vivo* experiment. C57BL/6J mice were inoculated with subcutaneous U14 tumors in the thigh on day 1. TGF-βi or PGE_2_i was administered intragastrically on days 14 and 15. RT was conducted on day 14 and Fluc^+^ DCs were injected intra-tumorally on day 16. Migration dynamics of Fluc^+^ DCs were traced over the subsequent 2 d. Tumor-draining LNs (t-LNs) were dissected 7 d after injecting Fluc^+^ DCs. Cell proliferation and T-cell priming in t-LN suspensions were evaluated. (E) A photo of the tumor site. (F) The effects of TGF-β and PGE_2_ on homing of DCs to t-LNs were investigated by bioluminescence imaging. (G) Cell number in the t-LN cell suspensions. (H-J) Results for T cell activation based on measurements of CD44 (H), CD69 (I), and TNF-α (J) expression in CD8^+^ T cells; N = 3; *p < 0.05. The above experiments were repeated twice.

An *in vitro* DC-T cell co-culture system was used to assess the impact of hypo-RT, TGF-β, and PGE_2_ on DC-T cell interaction (Fig. 6A). We observed that tumor secretions significantly impaired the expression of CD25, CD44, and CD107a in T cells (Fig. 6B and C), indicating their suppressive effect. Remarkably, when TGF-β signaling was blocked in combination with hypo-RT, this suppression was alleviated to a significant extent. These findings suggest that modulating DCs through the combination of hypo-RT and TGF-β inhibition, without blocking PGE_2_, can effectively promote DC activation of CD8^+^ T cells.

Next, a tumor-bearing mice model (Fig. 6D and E) was constructed to examine whether hypo-RT combined with TGF-β inhibition would enhance *in vivo* T cell priming. Fluc^+^ DCs were intra-tumorally injected and their migration to tumor-draining LNs (t-LNs) was examined. As depicted in Fig. 6F, the group receiving hypofractionated RT (20 Gy) combined with TGF-β blockade exhibited a substantial migration of DCs to t-LNs. However, when PGE_2_ was blocked, this enhancement was attenuated. The proliferation and activation of CD8^+^ T cells in t-LNs were consistent with DC homing. The 20 Gy/TGF-βi group exhibited the highest number of CD8^+^ T cells, along with the highest expression of CD44, CD69, and TNF-α (Fig. 6G-J). These results show that the combination of hypo-RT with TGF-β/PGE_2_ rebalancing can significantly enhance the migration of DCs to t-LNs. Moreover, this enhanced migration is correlated with increased CD8^+^ T cell proliferation and activation in t-LNs.

### Rebalancing TGF-β/PGE_2_ contributed to the therapeutic efficacy of hypo-RT

**Figure 7:**
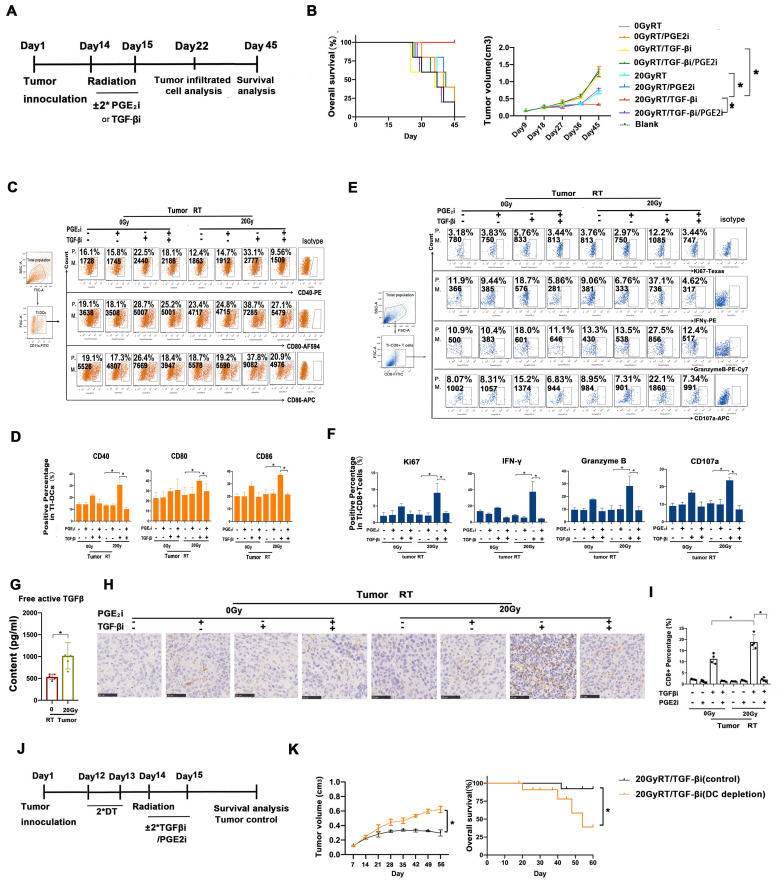
Rebalancing TGF-β/PGE_2_ contributed to the therapeutic efficacy of hypo-RT. (A) Flowchart of the establishment of the U14 tumor-bearing mouse model. Mice were treated with RT ± orally administered TGFβi/PGE_2_i. Seven days later, half of the mice were killed and tumor tissues were isolated and digested using type 4 collagenase. DC function and T cell activation in the tumor-infiltrated lymphocytes were detected. The remaining mice were maintained until day 45 at which point the survival rate and regression of tumors were evaluated. (B) Tumor growth and overall survival of tumor-bearing mice that received RT/TGFβi/PGE_2_i were evaluated; N = 5 in each group; *p < 0.05. A representative result from two repeats. (C-F) DC function (CD40, CD80, CD86) and T activation (IFN-γ, granzyme B, CD107, and Ki67) of tumor infiltrated lymphocytes were detected by FACS; *p < 0.05; P., positive percentage; M., mean fluorescence intensity. (G) Free active TGF-β in the TME was evaluated by ELISA; N = 5 in each group; *p < 0.05. (H-I) Immunohistochemical staining and quantification of CD8^+^ T cells in tumor samples from tumor-bearing mice that received RT/TGF-βi/PGE_2_i. Blind quantitation was done by the investigator utilizing Image J software; N = 4, *p < 0.05. (J) Flowchart of a diphtheria toxin (DT)-induced DC depletion experiment conducted to investigate the role of DCs in tumor control. (K) Tumor growth and overall survival of tumor-bearing mice with or without DC depletion that received RT combined with TGF-βi; N = 5 in each group; *p < 0.05. A representative result from two repeats.

To assess whether modulating TGF-β/PGE_2_ could enhance the therapeutic efficacy of hypo-RT, tumor control and prognosis were examined (Fig. 7A). Survival analysis demonstrated that mice in the 20 Gy RT/TGF-βi group had the longest lifespan and the slowest tumor growth (Fig. 7B). Moreover, flow cytometry analysis of tumor-infiltrating lymphocytes revealed that the greatest extent of DC maturation and CD8^+^ T cell activation was in this group (Fig. 7C-F). A high level of active TGF-β in *tumor lysis*, especially its upregulation after hypo-RT, was observed (Fig. 7G), supporting the *rationale* for its blockade. The infiltration of CD8^+^ T cells in tumors after various treatments (Fig. 7H-I) is consistent with the observed T cell activation (Fig. 7F). Furthermore, to confirm the essential role of endogenous DCs in tumor regression, we used CD11c-DTR/GFP transgenic mice, which exhibit a depletion of CD11c^+^ DCs when treated with diphtheria toxin. These mice were inoculated with U14 cells to produce subcutaneous tumors and then received the combined treatment of 20 Gy RT/ TGF-βi (Fig. 7J). DC depletion by diphtheria toxin significantly compromised tumor regression and reduced overall survival of the mice (Fig. 7K), confirming that DCs are indeed important targets for regulation by hypo-RT and TGF-β treatment. Collectively, our preclinical data highlight the critical role of the TGF-β/PGE_2_ cytokine balance in regulating DC maturation, migration, and DC-T cell activation within the post-RT TME, ultimately leading to tumor shrinkage.

**Figure 8:**
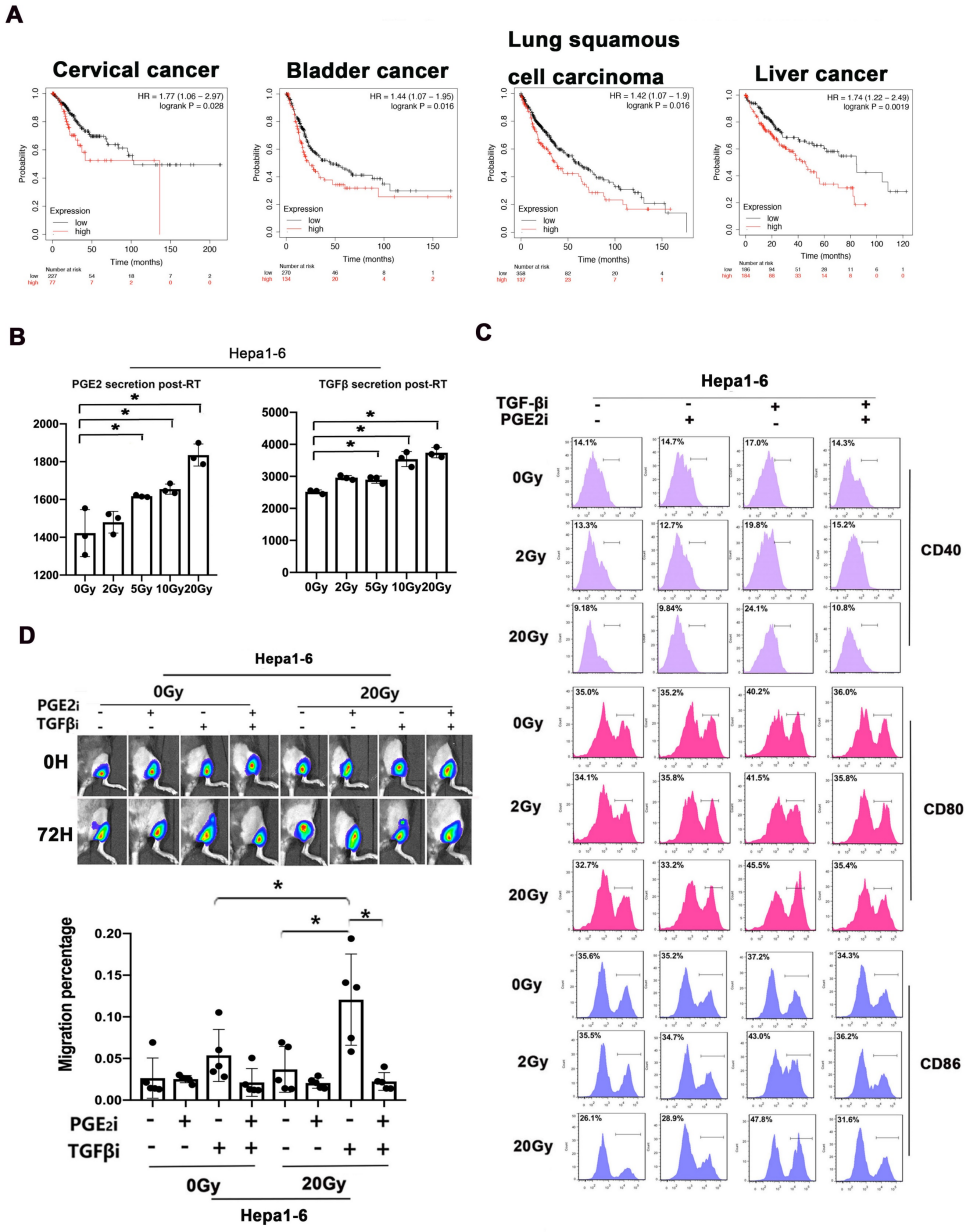
TGF-β/PGE_2_ imbalance post-RT immobilized DCs within liver tumors and worsened prognosis. (A) Bioinformatic analysis concerning the prognostic significance (overall survival) of the *TGFB1*/*COX2* gene ratio in different types of human cancers based on the Kaplan-Meier plotter database. (B) The secretion levels of TGF-β and PGE_2_ in the supernatant of irradiated Hepa1-6 cells based on ELISA; N = 3; *p < 0.05. (C) The expression of CD40, CD80, and CD86 in CD11c^+^ cells was detected using flow cytometry. (D) The effects of TGF-β/PGE_2_ balance on homing of DCs to tumor-draining LNs determined using bioluminescence imaging. N = 5 in each group; *p < 0.05. The above data are representative results from two independent experiments.

We analyzed the prognostic value of TGF-β/PGE_2_ balance in human cancer samples based on the Kaplan-Meier plotter database 27. We used the cyclooxygenase 2 (*COX2*) gene for analyzing the synthesis of PGE_2_ (Fig. 8A). Results showed that relatively low TGF-β and high PGE_2_ (low *TGFB1*/*COX2* gene ratio) was correlated with better overall survival in cervical cancer cohorts compared with high TGF-β and low PGE_2_ (high *TGFB1*/*COX2* gene ratio). Additionally, this prognostic effect of TGF-β/PGE_2_ balance was also observed in other tumor types including bladder cancer, lung squamous carcinoma, and hepatocellular cancer. Furthermore, we established a liver cancer mouse model utilizing Hepa1-6 cells and used it to confirm the importance of RT-induced TGF-β/PGE_2_ balance in DC homeostasis on other pathological types. PGE_2_ and TGF-β secreted by Hepa1-6 cells were upregulated by RT (Fig. 8B). DCs also acquired a suppressive phenotype in the presence of TGF-β (Fig. 8C). Importantly, hypo-RT combined with rebalancing TGF-β/PGE_2_ signaling successfully promoted liver tumor-infiltrated DCs homing to t-LNs (Fig. 8D). Collectively, these data suggested that the phenomena observed in cervical cancer can also be partially recapitulated in other cancer types, such as liver cancer, although substantial confirmatory experiments are still required.

## Discussion

RT, especially hypo-RT, primes the immune system against cancer cells via immunogenic cell death but remains constrained by the enhanced activity of suppressive immune cells. Key events, including changes in immunostimulatory cytokines, DC maturation/activation, and T cell recruitment and stimulation, highlight the potential of RT to prime potent responses against tumor cells if critical immunosuppressive effects can be overcome. Overcoming these effects to harness the potential of the immune system against cancer cells may result in beneficial local and abscopal effects 28. Indeed, preclinical studies have revealed an array of immune changes following RT that may affect the balance between antitumoral effects and tumor-promoting immunosuppression. The effectiveness of the radiation-induced anti-tumor immune response depends on the balance between immunostimulatory and immunosuppressive effects. However, shifting this balance to obtain survival benefits in clinical settings has rarely been achieved, which underscores the need for a better understanding of the interactions between RT and immunity 29.

There has been growing interest in enhancing the anti-tumor immune response by adjusting RT fractionation protocols (dose, fractionation, and timing) and the combined use of different immune modulators to activate the immune system 29. Most investigations about RT-immune modulator combined therapy focus on the activation and function of various T cell subpopulations, while their effect on DCs is often a point that is overlooked. It has been demonstrated that DCs maturation defines the immunological responsiveness of tumors to RT 30. Additional studies are needed to identify the specific factors and signaling pathways within various tumors that prevent DC maturation after treatment to improve responses to radiation and the efficacy of combination therapy. This study aimed to identify key mediators in the post-RT TME involved in DC dysfunction. We demonstrated for the first time that manipulating TGF-β/PGE_2_ balance combined with hypo-RT was beneficial in re-establishing DC maturation, homing to LNs, and cross-priming of T cells, which consequently could magnify the anti-tumor immune response.

DCs are sentinels of the immune system that migrate to the surrounding LNs, cross-present antigens to T cells, and induce the proliferation of tumor-specific cytotoxic T cells. Thus, the ability and magnitude of DC homing to T cell-enriched areas is a key limiting step for triggering anti-tumor responses 31. A non-invasive imaging technology was employed to map the dynamic migration of DCs *in vivo*. We propose that the secretion of TGF-β and PGE_2_ by irradiated cancer cells immobilizes DCs via MLC phosphorylation-mediated cytoskeletal reorganization and chemotaxis. The balance between TGF-β and PGE_2_ signaling in the TME arising from irradiation, can be adjusted to reboot DCs, which can promote T cell maturation in draining LNs, and bring about a series of subsequent positive effects, including improved T cell infiltration and increasing tumor shrinkage. PGE_2_ has been reported to support the induction of fully mature DCs capable of homing to LNs and is highly effective for priming naïve T cells 32. Therefore, PGE_2_ is a key component of the cytokine cocktail in adoptive DC therapy 33. PGE_2_ and its receptor regulate actomyosin contractility 34. The MLC signaling pathway is involved in the PGE_2_-induced rearrangement of actin in DCs. However, it remains unclear why the presence of TGF-β inhibits the activation of downstream PGE_2_ signaling pathways in DCs. One possible explanation is that considerable cross-talk may occur between the TGF-β and PGE_2_ intracellular signaling pathways, but this requires further investigation.

It has been shown that tumor-derived TGF-β inhibits the migration of DCs from tumors to their draining LNs, and that this immunosuppressive effect of TGF-β increases the risk of metastasis in the affected nodes 35. We found that TGF-β drives DCs toward tolerogenic polarization, and its blockade enhances DC polarization toward an immunogenic phenotype, which is consistent with the literature. However, our study also adds to the existing literature. First, we emphasize the key role of TGF-β/PGE_2_ cytokine balance in the regulation of DC function within the post-RT TME. Irradiation-induced upregulation of TGF-β strongly impaired the functionality of DCs. Conversely, PGE_2_ acted as an important positive modulator of the DC phenotype under TGF-β blockade. Against the background of TGF-β and PGE_2_ cytokine profiles altered by radiation, blocking TGF-β signaling while preserving PGE_2_ may be necessary to overcome RT-related immunosuppressive effects on DC maturation and migration. Second, this study sheds light on the application of TGF-β inhibitors in cervical cancer, revealing preclinical findings that could lead eventually to clinical trials focused specifically on cervical cancer. Finally, this study supports the significance of the dose radiation during RT combined with immunotherapy. Especially when conducting hypo-RT, the balance of TGF-β/PGE_2_ should not be ignored, which indeed affects the tumor control effect by impairing DC maturation and migration.

Immunogenic cell death is more common after exposure to higher-dose RT. Compared with other tumor types, the RT regimens of cervical cancer (external beam RT plus brachytherapy) could achieve higher biologically equivalent doses. Besides, for cervical cancer, the anatomical location provides the possibility of local direct medication, which potentially limits off-target effects. Thus, our findings have potential implications for clinical translation and modulation of TGF-β/PGE_2_ cytokine balance during RT in cervical cancer treatment. Currently, more than 10 drugs targeting the TGF-β signal pathway are now entering the clinic, including M7824, Galunisertib, and STP705. These drugs have been explored for use in pancreatic, gastric, colorectal, and hepatic cancers, and many other types of tumors. TGF-β targeting therapy is comparatively less explored in cervical cancer. These findings deepen our understanding of cervical cancer immunology, could promote the translation of TGF-β-targeting therapy for cervical cancer, and help facilitate the optimization of cervical cancer treatment strategies in the future.

Several key issues still need to be addressed in our study. First, we did not perform fractionated RT experiments in order to simplify the experiments and focus on cytokine balance. Therefore, there may be a gap between our findings and clinical practice. Further studies are necessary to determine the optimal fractionated regimen and whether ablative doses (biologically equivalent doses > 100 Gy) are required. Second, while we used galunisertib as the TGF-β signal inhibitor in this study, it is worth considering other safer and more cost-effective small-molecule drugs, such as losartan, which is already widely prescribed as an anti-hypertensive agent. Losartan has been shown to suppress TGF-β signaling and holds promise for translation to clinical application. Future studies should pay special attention to exploring the potential of such drugs. Finally, the prognostic significance of the TGF-β/PGE_2_ ratio in different cancer types was supported by bioinformatic data. A low TGF-β/PGE_2_ ratio was found to correlate with better overall survival in bladder cancer, lung squamous carcinoma, and hepatocellular carcinoma. While our findings may have implications for these specific cancer types, further experimental validation is required.

The overall process and major findings of our study are summarized in Fig. 8. We observed impaired DC function in the TME following hypo-RT, including reduced expression of allostimulatory molecules, decreased cytokine production, suppressed migration, and impaired T cell priming. However, by manipulating the TGF-β/PGE_2_ balance towards PGE_2_ polarization, we were able to restore DC function and facilitate the generation of effective anti-tumor responses. This work not only contributes to the rationalization of soon to be tested TGF-β-based combination immunotherapies against cervical tumors but also provides guidance for optimizing RT regimens in the future.

## Materials and Methods

### Mouse and cell lines

Female C57BL/6J mice 6-8 weeks-old were purchased from Charles River (Beijing, China, RRID: IMSR_JAX:000664) and housed at the Academy of Military Medical Sciences. L2G85 (FVB) mice (MSR_JAX:010548) with firefly luciferase (Fluc) were backcrossed with C57BL/6J mice (L2G85.C57BL/6 J) for seven generations. B6.FVB-1700016L21RikTg^(Itgax-DTR/EGFP)57Lan^/J(CD11c-DTR/GFP,IMSR_JAX:004509) transgenic mice were purchased from the Jackson Laboratory (Bar Harbor, ME). All procedures were approved by the Committee on Animal Care and Use of the Academy of Military Medical Sciences (approval number: IACUC-DWZX-2021-603). Mice were maintained according to the guidelines of the National Institutes of Health (USA). All procedures on mice were randomized and blinded. The cervical cancer cell lines U14 (RRID: CVCL 9U56) and SiHa (RRID: CVCL 0032) and the Hepa1-6 hepatoma cell line were purchased from Feiouer Biological Technology (Chengdu, China) and cultured in RPMI1640 medium supplemented with 10% fetal bovine serum (FBS).

### Radiation treatment

A medical linear accelerator (TrueBeam, Varian, Palo Alto, CA) was used to irradiate cells and tumor-bearing mice. The gantry was set at an angle of 180° to irradiate U14, Hepa1-6, and SiHa cells. A water phantom at a depth of 1.5 cm was used to form a dose-build-up area according to the dosimetric characteristics of the 6 Mv X-ray. Four to five layers of tissue bolus were placed on the surface of the culture plate to form a backscatter. The source to skin distance was 100 cm. The dose rate was 400 cGy/min Supplementary Fig 6 with radiation delivered at 2, 5, 10, and 20 Gy, respectively.

Tumor-directed radiation was delivered as a 20 Gy × 1 fraction regimen. Because the tumor diameters were less than 12 mm, low energy electron beams with small applicators and conformal lead blocks were used. Irradiation was performed on anesthetized mice using at a dose rate of 600 cGy/min. Each mouse was supinely placed on the couch of the linear accelerator and tumor-directed radiation was applied using lead shielding with an opening that exposed the tumor-bearing flank of the mouse.

### Induction of mouse bone marrow-derived immature DCs

Bone marrow cells were isolated from the femurs and tibias of wild-type C57BL/6J mice or L2G85.C57BL/6J transgenic mice to obtain wild-type DCs or Fluc+ DCs. Human DCs were derived from peripheral monocytes. Mouse bone marrow cells or human monocytes were resuspended in RPMI-1640 complete medium (containing 10% FBS, 5 ng/mL IL-4, and 10 ng/mL GM-CSF) at a density of 2 × 10^6^ cells/mL in 6-well plates. The medium was replaced with 2 mL of fresh medium containing GM-CSF and IL-4 added on day 3 and 5. The cells were used as immature DCs (imDCs) on day 6. Antibodies against CD11c-PE (Miltenyi Biotec, Westphalia, Germany, RRID: AB_2660154), CD11b-APC (Miltenyi Biotec, RRID: AB_2654646), CD45-APC-Cy7 (Thermo Fisher Scientific, Waltham, MA, RRID: AB_2534409), and MHCII-FITC (Miltenyi Biotec, RRID: AB_2802055) were used to detect the purity of DCs, with the results indicating that DCs accounted for almost 90% of the total population Supplementary Fig. 7A). The morphology of cultured imDCs was confirmed by microscopy (Supplementary Fig. 7B).

### DC phenotyping

Irradiated tumor cells were incubated for 24 h, and the supernatant was collected. Half of the supernatant was used to detect the secretion of TGF-β (DAYOU, 1217102), free active TGF-β (BioLegend, San Diego, CA, 437707), PGE2 (Elabscience, Houston, TX, E-EL-0034C), ATP (Promega Biotechnology Company, Madison, WI, F2000), and HMGB1 (Jianglai Biotech, JL51819-48T). The other half of the supernatant was co-cultured with imDC for another 24 h with or without added TGF-β receptor antagonist (TGF-βi: galunisertib, 50 μM) and PGE_2_ receptor antagonist (PGE_2_i: AH6809, 50 μM). The expression levels of CD40, CD80, CD86, MHCI, and MHCII in CD11c-positive cells were determined by flow cytometry. The DC suspensions were dispensed into 1.5 mL sample tubes and washed twice with 1 mL PBS (centrifuged at 1000 rpm for 10 min between washes). Cells were then resuspended in 100 μL staining buffer and fluorescent antibody to stain APC-CD11c (BioLegend, 117309, RRID: AB_313778) combined with PerCP-Cy5.5-H-2Kb (BioLegend; 116515, RRID: AB_1967107), PE-CD40 (BioLegend, 157505, RRID: AB_2832552), FITC-CD80 (BioLegend, 104705, RRID: AB_313126) or PerCP-Cy5.5-CD86 (BioLegend, 105027, RRID: AB_89342). To detect the chemokine receptors on DCs, cells were processed as described and the APC-CD11c antibody combined with PE-CCR2 (BioLegend, 150609, RRID: AB_2616981), PE-CXCR4 (BioLegend, 153805, RRID: AB_2734225), PE-CCR5 (BioLegend, 107005, RRID: AB_313300) or PE/Cyanine7-CCR7 (BioLegend, 120123, RRID: AB_2616687). These were mixed at room temperature for 15 min in the dark and then washed twice with PBS. The double-labeled samples were subjected to FACS analysis in an 8-color FACS Calibur flow cytometer (BD Biosciences, Mountain View, CA). Data for 10,000 cells were collected and analyzed using Flow Jo 7.6.1 software (FlowJo, RRID: SCR_008520). Three or four replicates of each experiment were conducted.

### Secretion of proinflammatory factor by DCs

Irradiated tumor cells were incubated for 24 h, and the supernatant was collected. ImDCs were incubated with the supernatant for another 24 h with or without the TGF-β and PGE_2_ receptor antagonists, galunisertib (50 μM) and AH6809 (50 μM), respectively. The supernatant was collected by centrifugation at 1000 rpm for 10 min and stored in a 1.5 mL tube. The concentrations of IL-12p70 (DAYOU, 1211202), IL-6 (Reddot Biotech, Kelowna, Canada, RD-IL6-Mu-24T), IL-1β (DAYOU, 1210122) and TNF-α (DAYOU, 1217202) were measured according to the instructions of the ELISA kit. The experiments were done in triplicate.

### Immunofluorescence of cytoskeletal organization

DCs were seeded onto a microscope slide and co-incubated for 24 h with supernatant from tumor cells. Thereafter, the cells were fixed at room temperature for 15 min with 4% paraformaldehyde. The slide was rinsed three times with 1 × PBS to remove paraformaldehyde and then permeabilized with 0.1% Triton X-100 in PBS for 2 min at room temperature. For actin staining, permeabilized cells were stained with 300 μL phalloidin (Abcam, Cambridge, United Kingdom, ab176753, 50 μg/mL) for 1 h at room temperature. For β-tubulin staining, permeabilized DCs were labeled with anti-β-tubulin (LSBio, Lynnwood, WA, LS-C136798-100, RRID: AB_10948667, 10 μg/mL) overnight, followed by washing and addition of Cy3-goat anti-mouse IgG (2 μg/mL) for 2 h at room temperature. After washing, all microscope slides were mounted with DAPI and viewed by confocal laser-scanning microscopy (Zeiss, LSM510META). Immunofluorescence analysis was done using Volocity 3D Image Analysis Software (Quorom Technologies, Puslinch, Canada, RRID: SCR_002668) by an investigator blinded to the identity of the slides.

### Analysis of DC homing and *in vivo* T cell activation

To detect DC homing to local LNs, irradiated tumor cells were incubated for 24 h, and the supernatant was collected. Fluc+ imDCs from L2G85.C57BL/6 mice were incubated with supernatant for another 24 h with or without adding TGF-β and PGE_2_ receptor antagonists. The DCs were collected and injected into the footpad of wild-type C57BL/6J mice (1×10^6^ DCs per mouse) for dynamic imaging. To detect the distribution of circulating DCs, 3 × 10^6^ Fluc+ DCs were intravenously injected into mice via the tail vein and photographed at 0 and 48 h post-injection. To evaluate intra-tumoral DC homing to tumor-draining LNs, C57BL/6J mice were subcutaneously inoculated with 2 × 10^6^ U14 cells or Hepa1-6 cells into the thigh on day 1. When tumors were 5 mm in average diameter, mice were randomized to receive RT (on day 14) and intragastrically administered TGF-βi/PGE_2_i (TGF-βi: galunisertib, 75 mg/kg; PGE_2_i: AH6809, 30 mg/kg, on days 14 and 15). Then 1 × 10^5^ Fluc+ DCs were intra-tumorally injected (on day 16). DC homing to the draining LN was traced for the subsequent 2 d using the IVIS Spectrum system (PerkinElmer, Inc., Waltham, MA). Seven days after intra-tumoral DC injection (i.e., day 23), the tumor-draining ILN was isolated and thoroughly ground for analysis of *in vivo* T cell activation. The total number of lymphocytes from the ILN was calculated using a cell counter. Subsequently, the percentage of CD44, CD69, and intracellular TNF-α of CD8α-positive cells were co-labeled with FITC-CD8ɑ (BioLegend, 100705, RRID: AB_312744) and PE-CD69 (BioLegend, 104507, RRID: AB_313110), APC-TNF-α (BioLegend, 506307, RRID: AB_315428) or APC-CD44 (BioLegend, 103011, RRID: AB_312962) and then detected by flow cytometry. The single-cell suspension was divided into two-sample tubes. The cells in tube 1 were washed once with 1 mL of PBS (1000 rpm for 10 min) and then resuspended in 100 μL of staining buffer. The fluorescent antibodies FITC-CD8ɑ, PE-CD69, and APC-CD44 were added followed by incubation at room temperature for 15 minutes in the dark. The cells were washed twice with 1 mL of PBS (1000 rpm for 10 min between washes) and resuspended in 300 μL of PBS for flow cytometry. Cells in tube 2 were ruptured with fixation/permeabilization reagent, followed by addition of FITC-CD8ɑ and APC-TNF-α antibodies. Cells were resuspended in 1 × BD Perm/Wash^TM^ buffer (BD Biosciences) for flow cytometry.

### Western blotting analysis

The supernatant of tumor cells was incubated with DCs as previously described. Changes in MLC protein levels in DCs in response to different treatments was determined using western blotting analysis. The antibodies used were anti-MLC (Abcam, ab76092, RRID: AB_1524000), anti-pMLC (Abcam, ab157747), and anti-β-actin (Abcam, ab8226, RRID: AB_306371). The dilution ratio of pMLC and MLC antibodies was 1/1000. Total cellular protein was separated by sodium dodecyl sulfate-polyacrylamide gel electrophoresis on 10% gels and transferred to polyvinylidene difluoride membranes. The membranes were incubated with primary antibodies overnight, followed by the corresponding secondary antibodies for 1 h at room temperature. β-Actin was used as a loading control. The signals were detected using an ECL western blotting detection kit (Abcam, ab133408).

### Immunohistochemical staining

The tissue submitted for immunohistochemistry was a representative site of the cervical intra-tumoral area. Specimens were deparaffinized in a 65 ℃ oven for 10 min and then immersed in consecutive baths of 100%, 90%, 80%, and 60% alcohol for 8 min for rehydration. Endogenous peroxidase activity was blocked with 3% H_2_O_2_ for 15 min. Consecutive tissue slices were autoclaved (140 ℃) in EDTA (pH 8) for 3 min. Tissue slides were incubated with primary antibodies for CD8ɑ (1:100 diluted, Abcam, ab217344, RRID: AB_2890649), CD11c (1:100 diluted, Abcam, ab195534, RRID: AB _844236), and TGF-βI (1:50 diluted, Abcam, ab170874, RRID: AB_2895231) were at 4 ℃ in a refrigerator overnight. Afterward, the slides were incubated with a corresponding HRP-labeled secondary antibody (1:250) using EnVision System reagents (Agilent Technologies, Santa Clara, CA) for 20 min at room temperature. Then, they were stained with DAB for 3-5 mins in a wet chamber and counterstained with hematoxylin for 8-10 s. Later, the slides were dehydrated in alcohol and mounted with coverslips. The wash buffer was PBS at all stages. For counting positive staining cells, immunohistochemistry sections were first evaluated at low power (100×), and six representative areas where TGF-β and CD11c were positively stained at high or low density were identified. The positive area was assessed using a semi-quantitative approach (36 utilizing Image J software (ImageJ, RRID: SCR_003070) for data analysis.

### Bioinformatic analysis

The TIMER database was used to systematically analyze the tumor-infiltrating immune cells in thirty-two tumor types using more than 10,000 samples from the TCGA database (https://cistrome.shinyapps.io/timer/) 37. We analyzed the association between the level of *TGFB1* gene expression and the abundance of dendritic cells in CESC and the maturation markers of dendritic cells. The mRNA levels of *TGFB1* in several cancers including cervical cancer were also identified from the TCGA RNA-seq data using the TIMER database.

Kaplan-Meier survival curve analysis was performed to assess the correlation between the expression of 54,000 genes and the survival rates in 21 different cancers using more than 10,000 cancer samples. In this study, Kaplan-Meier plots (http://kmplot.com/analysis/) were used to analyze the relationship between the ratio of the *TGFB1* and *COX2* genes (*TGFB1*/*COX2*) and survival rates in several types of cancers based on the hazard ratios and log-rank P-values. Cyclooxygenase 2 promotes the release of PGE_2_. Patients were sorted by auto selection of the best cutoff values. The optimal cutoff values are determined by automatic calculation and are available directly on the Kaplan*-*Meier diagram website. We provide the cutoff value used in the analysis Supplementary Table 2.

### Tumor inoculation and survival measurements

C57BL/6J wide type mice and CD11c-DTR/GFP transgenic mice were inoculated subcutaneously with U14 cells in the thigh on day 1. When tumors were 5 mm in average diameter, mice were randomized to receive RT on day 14 and TGF-βi or PGE_2_i intragastrically (TGF-βi: galunisertib, 75 mg/kg; PGE_2_i: AH6809, 30 mg/kg) on day 14 and day 15. At the same time, the CD11c-DTR/GFP transgenic mice received diphtheria toxin (two intraperitoneal injections, 4 ng DT/g body weight) to deplete DC populations. Tumor length and width were measured using calipers. Mice were euthanized when tumor size exceeded 12 mm in any dimension or when the body condition score declined significantly. Tumors were dissected and subjected to immunohistochemical staining with an anti-CD8ɑ antibody.

### Statistical analysis

**Figure 9:**
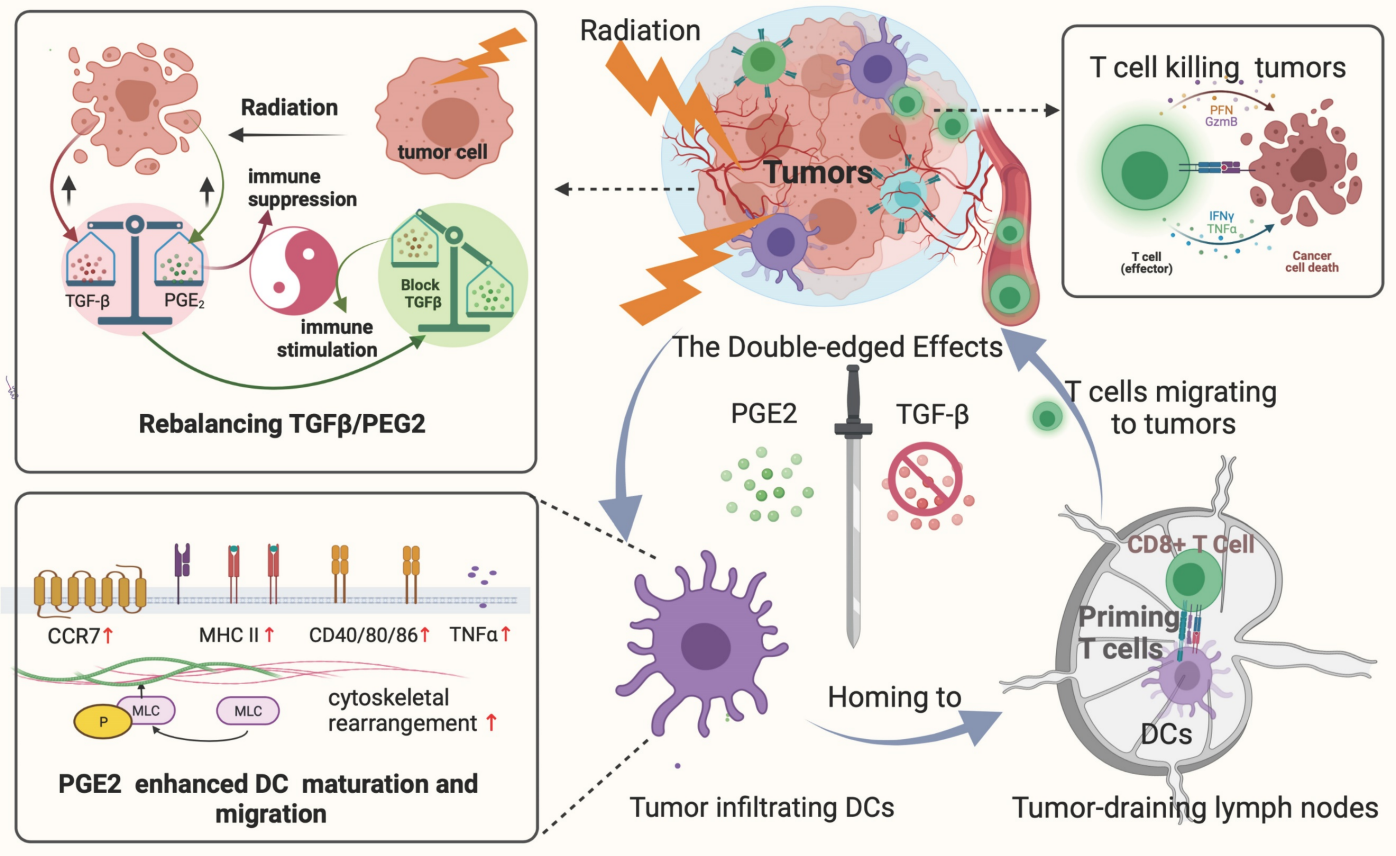
Rebalancing the TGF-β/PGE_2_ ratio could reverse RT-related immunosuppressive effects by rebooting DC maturation and homing.

The quantitative data are expressed in the form of mean ± SD. Comparisons were conducted using a two-tailed *t*-test, one-way ANOVA, or Mann-Whitney U test. A p-value < 0.05 was considered statistically significant. The GraphPad Prism 6 software was used for the graphing and statistical analysis. The schematic diagram in Figure 9 was drawn in BioRender (license number: WM2434XP73).

Other details about reagents and antibodies can be found in the supporting information.

## Supplementary Material

Supplementary figures and tables.
