# Supplementary material for: Rebalancing TGF-β/PGE2 breaks RT-induced immunosuppressive barriers by enhancing tumor-infiltrated dendritic cell homing
Source: Int J Biol Sci. 2024 Jan 1;20(1):367–86. doi: 10.7150/ijbs.87867 (PMC10750293; doi:10.7150/ijbs.87867)
Supplement: Supplementary file 1 — Supplementary figures and tables. [file ijbsv20p0367s1.pdf]

Figures in supporting information

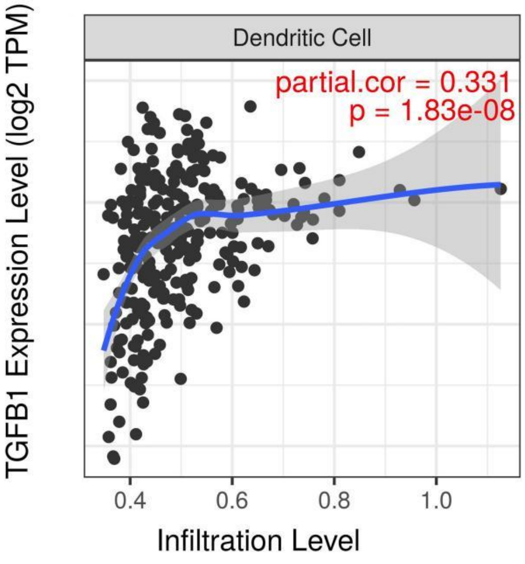

Sup Fig 1. Correlation analysis of TGFB1 expression and infiltration levels of DCs in cervical cancer tissues using the TIMER database.

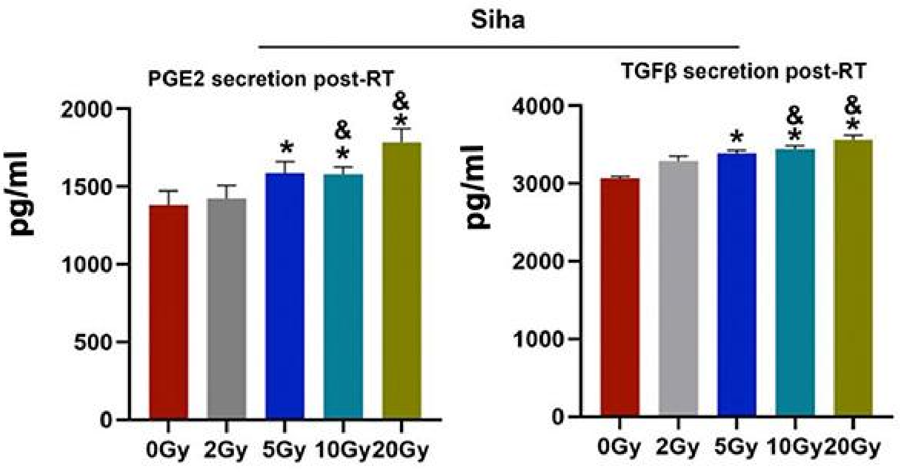

Sup Fig 2. The secretion level of TGF β and PGE_2 in the supernatant of radiated Siha by ELISA.(N=3,*compared with 0Gy group p<0.05.&compared with 2Gy group p<0.05).

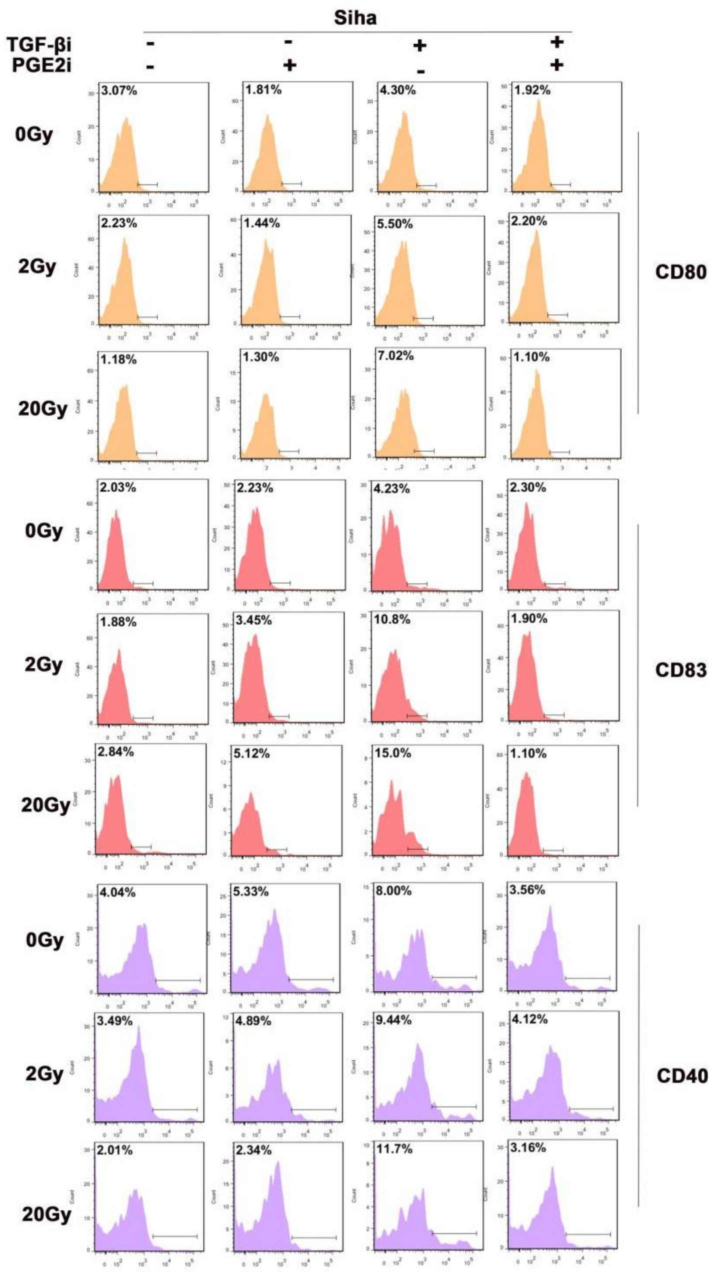

Sup Fig 3. RT exacerbated the suppressive effect of Siha cells on DCs. Blocking TGF-β can reverse the radiation-induced suppressive effect of Siha on DCs via PGE_2 secretion. The supernatant of radiated Siha was cocultured with human derived dendritic cells pretreated with TGF-βi/PGE_2i. 24 hours later DCs were stained with FITC-CD80, PE-CD83, PEcy7-CD40.

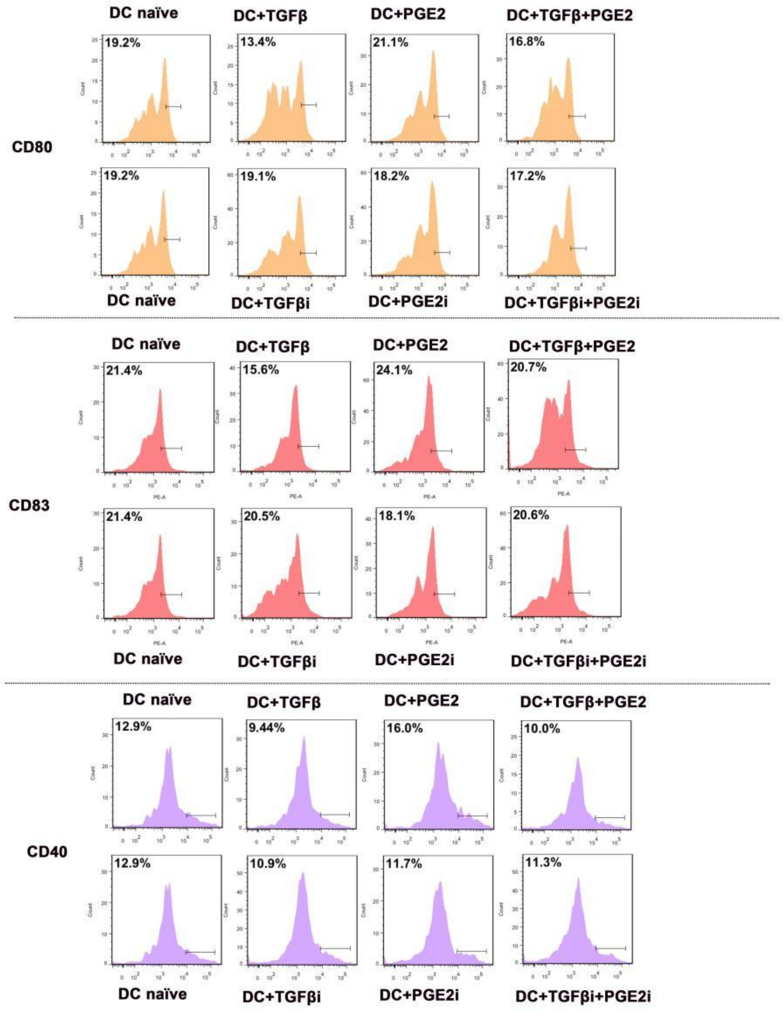

Sup Fig 4. Blocking both PGE2 and TGF-β directly on DC did not alter the functional phenotype significantly. Human-derived dendritic cells were pretreated with TGFβi, PGE2i,PGE2 and TGF β . 24 hours later DCs were stained with FITC-CD80, PE-CD83 and PECy7-CD40.

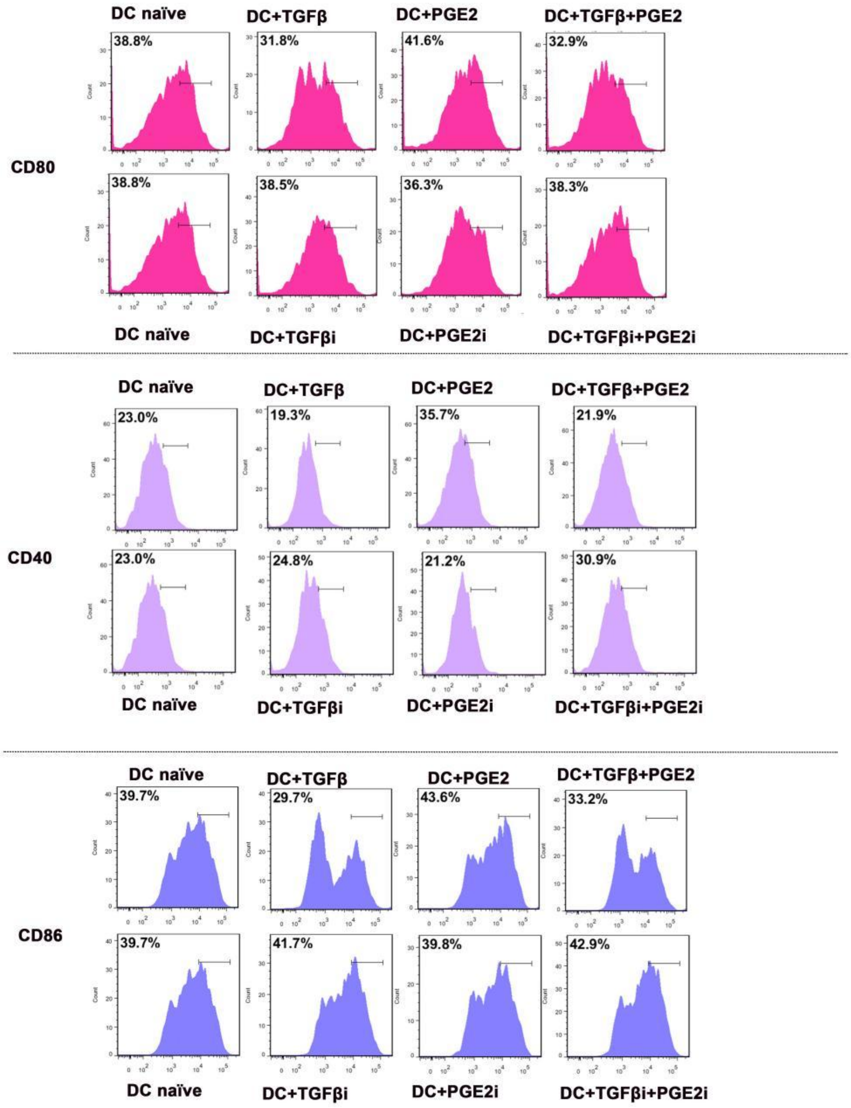

Sup Fig 5. Blocking both PGE2 and TGF-β signal directly on DC did not alter the functional phenotype significantly. Mouse-derived dendritic cells were pretreated with TGFβi, PGE2i, PGE2 and TGFβ. 24 hours later DCs were stained with PE-CD40, APC-CD80 and FITC-CD86.

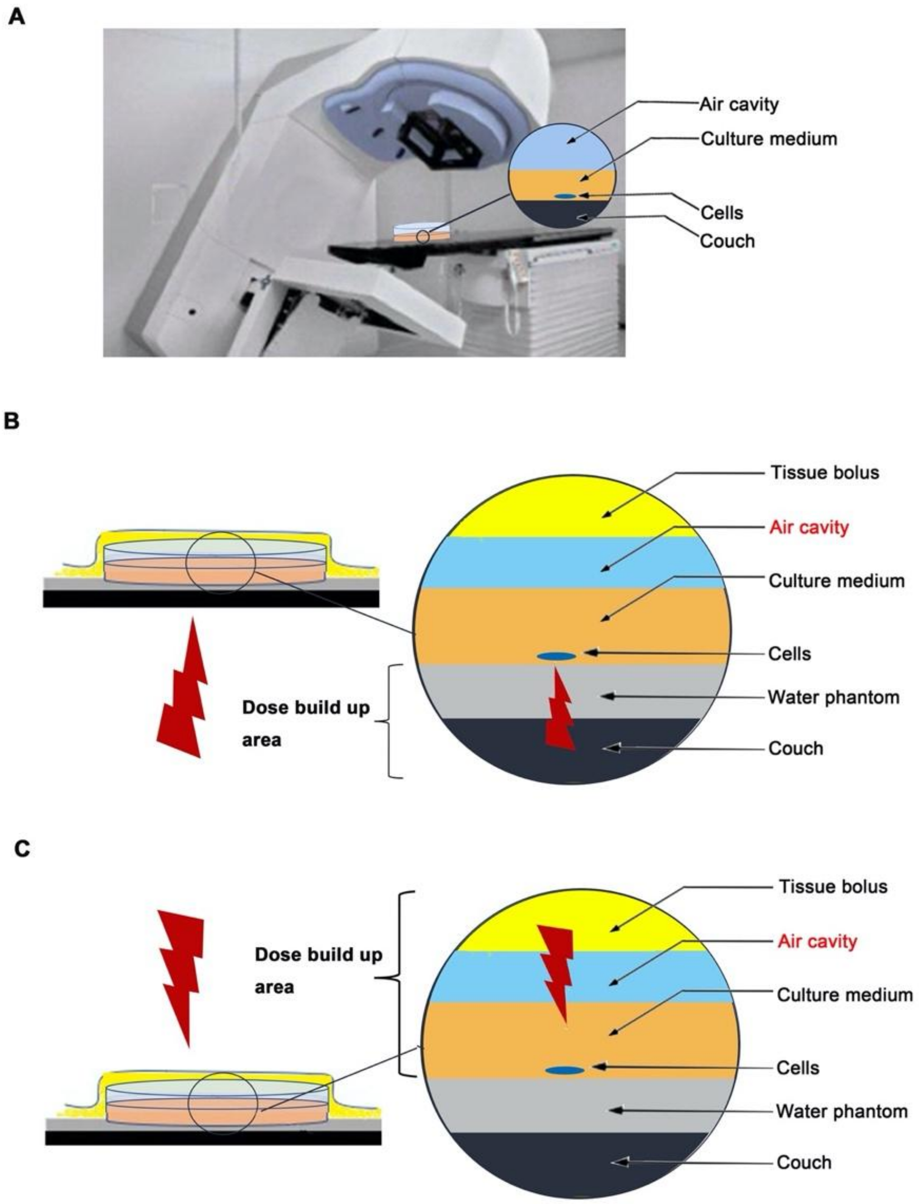

Sup Fig 6. Schematic diagram of cell irradiation details. (A) Rotate the gantry to the appropriate angle. (B) Irradiate the cells at an incidence angle of 180 ˚ , the photon beam will pass through the treatment bed (Couch), the water phantom and reach the cells in turn; (C)

Irradiate the cells at an incidence angle of 0 ˚ , the photon beam will pass through the tissue bolus, the air cavity and the culture medium and culture medium to reach the cells.

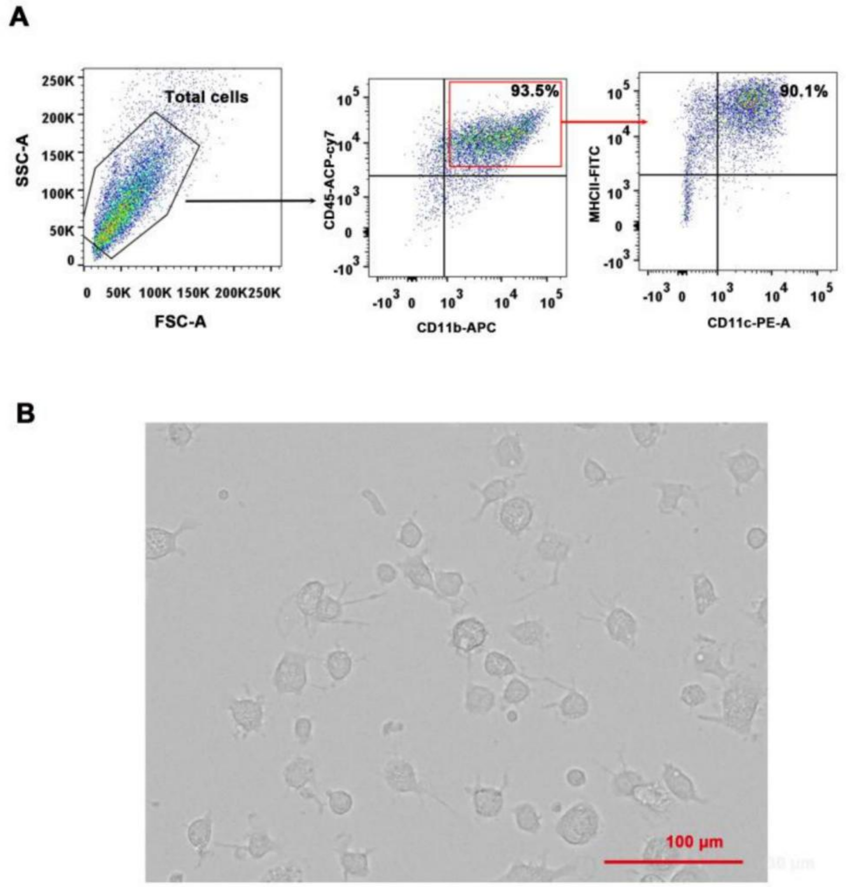

Sup Fig 7. Identify the bone marrow derived cells used in this research.

A)**.** DCs with combined antibodies of CD11c-PE, CD11b-APC, CD45-APC-Cy7, and MHCII-FITC were detected to show the purity of DCs we used. A sequential gating strategy was employed to identify DCs populations expressing CD11b+ CD11c+ CD45+ MHCII+ within the bone marrow derived clusters. **B)** Dendritic cell morphology of bone-marrow cultures is shown on days 5 for cultures supplemented with mouse GM-CSF/IL4. Images were obtained using optical camera(scale bars, 100 μm) which revealed that the vast majority of cells in the field of vision displayed the typical morphology of DCs.

Sup Table 1. The characteristics of cervical cancer patient involved in this analysis.

Patient No.	Tumor stage (FIGO)	Pathology Diagnosis	Date of radiotherapy begin	Radiation regimes	Date of radiotherapy complete	Date of surgery
1	IB2	High differentiated adenocarcinoma	2015-9-14	Pelvic IMRT: 50.4Gy/1.8Gy/28F 192Ir Brachytherapy: 36Gy/6Gy/6F	2015/11/5	2015/12/15
2	IIB	Mid differentiated adenocarcinomas	2015-4-7	Pelvic IMRT: 50.4Gy/1.8Gy/28F 192Ir Brachytherapy: 36Gy/6Gy/6F	2015/5/27	2015/7/14
3	IIB	Low differentiated adenocarcinomas	2010-1-18	Pelvic IMRT: 50.4Gy/1.8Gy/28F 192Ir Brachytherapy: 36Gy/6Gy/6F	2010/3/8	2010/5/29
4	IB2	High differentiated adenocarcinoma	2014-8-19	Pelvic IMRT: 41.4Gy/1.8Gy/23F 192Ir Brachytherapy: 30Gy/6Gy/5F	2014/9/30	2014/12/15
5	IIB	Low differentiated adenocarcinomas	2010/9/1	Pelvic IMRT: 50.4Gy/1.8Gy/28F 192Ir Brachytherapy: 36Gy/6Gy/6F	2010/10/12	2010/12/7
6	IB2	High differentiated adenocarcinoma	2014-11-20	Pelvic IMRT: 50.4Gy/1.8Gy/28F 192Ir Brachytherapy: 36Gy/6Gy/6F	2015/1/9	2015/6/26
7	IB2	Mid differentiated adenocarcinoma	2009-10-21	Pelvic Conventional RT 50Gy/2Gy/25F 192Ir Brachytherapy: 36Gy/6Gy/6F	2009/11/30	2010/2/23
8	IIB	Mid differentiated adenocarcinoma	2011-11-28	Pelvic IMRT: 50.4Gy/1.8Gy/28F 192Ir Brachytherapy: 36Gy/6Gy/6F	2012/2/13	2012/6/13

Sup Table 2. The details of bio-imformatic analysis on TGFB1/COX2 ratio and overal survival on the Kaplan-Meier diagram website.

Tumor type	Patient number	Cutoff value used in analysis	P values
Cervical cancer	304	0.03	0.028
Bladder cancer	404	0.02	0.016
Lung squamous cell carcinoma	501	0.05	0.016
Liver hepatocellular carcinoma	371	0	0.0019
